# Active Fault-Tolerant Control for Steering Actuator Bias in Autonomous Vehicles Using Adaptive Sliding Mode Observer

**DOI:** 10.3390/s26051680

**Published:** 2026-03-06

**Authors:** Hyunggyu Kim, Wongun Kim

**Affiliations:** 1 Specialized Machinery and Robotics Group, Korea Institute of Industrial Technology (KITECH), Gimje 54325, Republic of Korea; khg@kitech.re.kr; 2 Department of Electronic Information Engineering, Jeonbuk National University, Jeonju 54896, Republic of Korea

**Keywords:** steering actuator bias, fault detection and diagnosis, adaptive sliding mode observer, fault-tolerant control, autonomous vehicles, lateral vehicle dynamics, fault diagnosis and estimation

## Abstract

**Highlights:**

**What are the main findings?**

- A real-time steering actuator bias estimation method is developed using an adaptive sliding mode observer based on lateral vehicle dynamics
- Steering actuator bias faults are reliably detected and reconstructed under high-speed driving conditions without requiring additional sensors or hardware redundancy

**What are the implications of the main findings?**

- The proposed approach improves the reliability of steering actuator systems by enabling continuous monitoring and diagnosis of bias-type faults.
- The method supports fault-tolerant autonomous driving by providing accurate fault information that can be directly utilized for control compensation.

**Abstract:**

Autonomous vehicle path-tracking and lateral stability depend critically on reliable steering actuator operation. However, steering systems are susceptible to bias faults from mechanical misalignment, friction, drivetrain asymmetry, and degradation. These faults distort commanded versus actual steering inputs, causing accumulated lateral and heading errors during high-speed driving. Actuator biases manifest as constant offsets, gradual drift, or intermittent activations, which complicate reliable diagnosis. This study presents an adaptive sliding mode observer-based active fault-tolerant control framework for real-time detection, estimation, and mitigation. An extended four-state lateral error model incorporating distance and heading errors captures the influence of steering bias on vehicle behavior and stability. Adaptive observer gain tuning addresses modeling uncertainties arising from speed variations, linearization residuals, and tire stiffness changes to ensure robust estimation under realistic driving conditions. The effectiveness of the proposed method is validated through high-speed double lane change simulations considering three representative bias scenarios: an initial constant bias, a gradually increasing drift bias, and an intermittent bias. Results demonstrate reliable bias estimation and significantly improved path-tracking accuracy compared to uncompensated cases. Operating without additional sensors, hardware redundancies, or controller switching, the framework is suitable for practical implementation in autonomous vehicle steering systems.

## 1. Introduction

In autonomous vehicle systems, the integrity of the steering actuator is crucial for reliable lateral motion control because the steering input serves as the primary interface between control commands and vehicle dynamics. Steering actuator bias faults, which are commonly induced by long-term wear, calibration errors, or electronic drift, introduce a continuous offset in the applied steering angle without causing observable signal discontinuities. This fault characteristic makes bias-type actuator faults particularly challenging to detect because the measured signals may remain smooth and within nominal ranges, while the effective control input gets persistently distorted. Consequently, such faults can silently deteriorate path-tracking performance and vehicle attitude stability, thereby highlighting the need for robust fault reconstruction and active compensation strategies to mitigate steering actuator bias in real time without altering the baseline control architecture.

Fault diagnosis and fault-tolerant control (FTC) have been extensively investigated for safety-critical control systems, with numerous model-based approaches proposed to detect and compensate for actuator and sensor faults [1,2,3,4,5]. Among these approaches, observer-based techniques play a central role in fault estimation without relying on hardware redundancies. Particularly, sliding mode observer (SMO)-based methods have demonstrated strong robustness against uncertainties and disturbances, enabling the direct reconstruction of actuator faults through equivalent output error injection and their subsequent use in active FTC (AFTC) schemes [1,3,5]. Simultaneously, model-based fault diagnosis strategies using analytical redundancy relations or residual evaluations have been applied to detect actuator and sensor faults in vehicle steering systems [2,6]. However, most conventional SMO approaches utilize fixed switching gains to ensure robustness against worst-case bounded uncertainties. In practical autonomous driving scenarios, such high fixed gains often induce excessive chattering, which complicates the distinction between persistent and high-frequency road disturbances. Furthermore, existing steering-fault studies [2,4] rely on simplified dynamics, which may not fully capture the coupling effects between lateral deviation and heading error when a continuous bias distorts the steering input.

Building on this foundation, active fault-tolerant steering control strategies have been proposed by modeling actuator faults as a loss of effectiveness and compensating for them through adaptive controller reconfiguration or gain scheduling [4,7]. Alternatively, passive and reconfigurable fault-tolerant path-tracking controllers have been developed to enhance robustness against actuator or steering system faults, including differential steering-based approaches and prescribed performance control frameworks that exploit actuator redundancy [8,9,10]. However, the reliance on actuator redundancy is generally impractical for standard commercial vehicles equipped with single-steering systems. Furthermore, mechanism switching or controller reconfiguration strategies may introduce undesirable transient oscillations, making them less effective in compensating for continuous bias faults where smooth and seamless mitigation is required.

Comprehensive overviews of AFTC frameworks that integrate fault detection and compensation have been systematically reported in the literature, providing a broad perspective on fault diagnosis, estimation, and accommodation strategies in safety-critical systems [11]. In the automotive domain, FTC approaches have been extended to steer-by-wire systems where actuator faults are addressed through model-based estimations and reconfigurable control architectures [12]. Nevertheless, these frameworks typically assume fault information in predefined forms and do not emphasize the real-time reconstruction of the steering actuator bias for path-tracking control.

Recently, optimization-based approaches have been designed for fault-tolerant path tracking in autonomous vehicles. Model predictive control (MPC)-based frameworks have been proposed to mitigate the effects of sensor or actuator faults by incorporating fault estimation results into constraint-tightening or controller reconfiguration schemes [13,14]. Advanced MPC formulations have been developed to handle steering actuator faults in over-actuated vehicle platforms by exploiting additional control degrees of freedom [15,16]. Although effective, the heavy computational burden of online iterative optimization hinders its deployment in cost-effective automotive electronic control units (ECUs). Moreover, these methods often require the complete replacement of their existing control architecture, limiting their flexibility to lightweight add-on fault-tolerant modules.

Data-driven and learning-based methods have been explored for steering actuator fault diagnosis, in which deep neural networks are employed to detect or classify bias and effectiveness faults under noisy measurement conditions, often triggering auxiliary control modes such as direct yaw control [17,18]. AFTC strategies combining online fault estimation with predictive control have been reported to compensate for steering deviations and model mismatches through optimization-based uncertainty estimations [19]. Static output feedback and redundancy-based control schemes have been examined to tolerate steering actuator faults by redistributing control efforts among multiple actuators [20]. However, data-driven methods rely extensively on the quality and quantity of labeled training datasets, frequently failing to generalize to unseen fault scenarios. Furthermore, redundancy-based approaches are constrained by hardware availability, whereas a physically insightful observer-based approach directly reconstructs the bias using only onboard sensor measurements.

To address these limitations, this study proposes an adaptive SMO (ASMO)-based fault detection and an AFTC algorithm to achieve real-time mitigation of steering actuator bias faults in autonomous vehicles. To appropriately reflect the influence of steering bias on lateral vehicle motion and attitude stability, an extended four-state lateral error state-space model is developed, including the lateral distance and heading angle errors. Further, an ASMO is designed based on this model. Owing to an extended state representation, the error dynamics cannot be simplified into a single scalar term, thereby requiring a multi-injection structure that independently addresses each state error. Accordingly, the injection gains are designed in a vectorized form, enabling the construction of a robust sliding structure tailored to the sensitivity and dynamic characteristics of individual state variables.

The proposed observer estimates the magnitude of the steering actuator bias in real time by evaluating the discrepancy between the commanded steering input and the actual steering response. Further, the estimated bias is directly compensated at the output of the existing path-tracking controller, enabling an immediate fault-tolerant action without modifying the control structure or introducing controller switching. During this process, the slope of the sliding surface and the adaptive gains are carefully tuned to suppress chattering while preserving the convergence and robustness of the bias estimation. Consequently, the proposed method effectively maintains the path tracking accuracy and vehicle attitude stability, even in the presence of steering actuator bias faults, and can be seamlessly integrated into existing path-tracking systems as a lightweight add-on module.

The main contributions of this study are as follows: 1. Extension of the Lateral Error Model: An augmented four-state state-space model is formulated to explicitly account for the coupling between lateral distance and heading angle errors, providing richer information for fault reconstruction than conventional two degrees of freedom (2-DOF) models. 2. Adaptive Sliding Mode Observer Design: A novel adaptive gain-scheduling law is introduced to minimize the chattering phenomena while maintaining robust bias estimation performance against external disturbances. 3. Active Fault Compensation: The estimated bias is seamlessly integrated into the baseline path-tracking controller, allowing immediate fault compensation without requiring complex controller reconfiguration.

The remainder of this paper is organized as follows: Section 2 presents the software architecture of the autonomous vehicle system. Section 3 describes the lateral vehicle dynamics model and baseline path-tracking control algorithm. Section 4 details the design principles of the proposed steering actuator fault detection and AFTC algorithm. Section 5 evaluates the effectiveness of the proposed method through simulation studies under various steering actuator bias scenarios. Section 6 concludes the paper with a discussion of future research directions.

## 2. System Architecture and Simulation Environment

### 2.1. Overall Control Architecture

**Figure 1:**
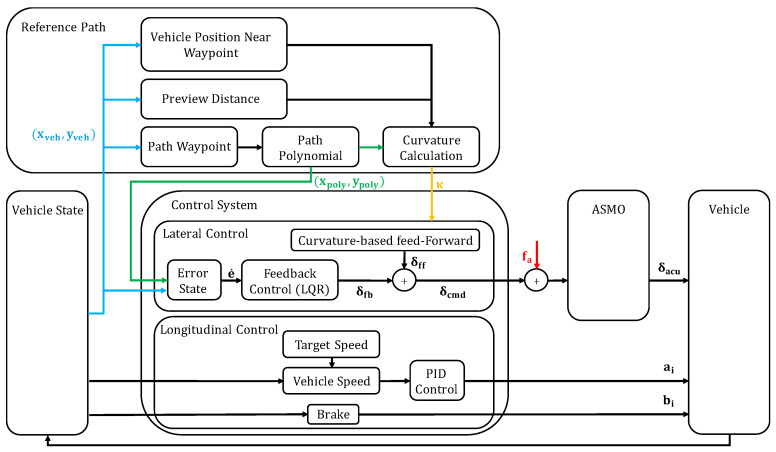
Overall system architecture of the proposed algorithm.

The overall architecture of the proposed FTC system is shown in Figure 1. The entire framework was implemented in the MATLAB/Simulink (R2023a) environment to validate the driving stability and path-tracking performance under steering actuator failure scenarios. The system comprises three main hierarchical layers: the reference path generator, the control system, and the vehicle plant.

As shown in Figure 1, a reference path is generated based on a set of waypoints. Adjacent waypoints are connected using high-order polynomials to create a smooth and continuous trajectory, from which the reference curvature (κ) can be calculated. The control system layer consists of a longitudinal controller (PID-based) for speed maintenance and a lateral controller. The lateral controller combines a linear quadratic regulator (LQR)-based feedback mechanism with a curvature-based feedforward term to minimize tracking errors. The vehicle plant module simulates the physical response of the vehicle to the steering and throttle inputs.

### 2.2. Actuator Fault Modeling

To verify the estimation performance of the proposed algorithm against steering actuator bias faults, the steering actuator is modeled as an ideal positioning device that follows the command input. This approach isolates the bias fault effect by directly introducing an additive bias ($f_{a}$) into the steering command channel as:

(1) $$\delta_{actual}\left(t\right)=\delta_{cmd}\left(t\right)+f_{a}(t),$$

where $\delta_{cmd}$ represents the nominal steering command computed by the controller and $\delta_{actual}$ denotes the distorted steering angle applied to the vehicle plant. The developed setup simulates a condition in which the mechanical steering system deviates from the electronic command owing to a sensor offset or mechanical deformation.

### 2.3. Fault Estimation and Compensation Scheme

**Figure 2:**
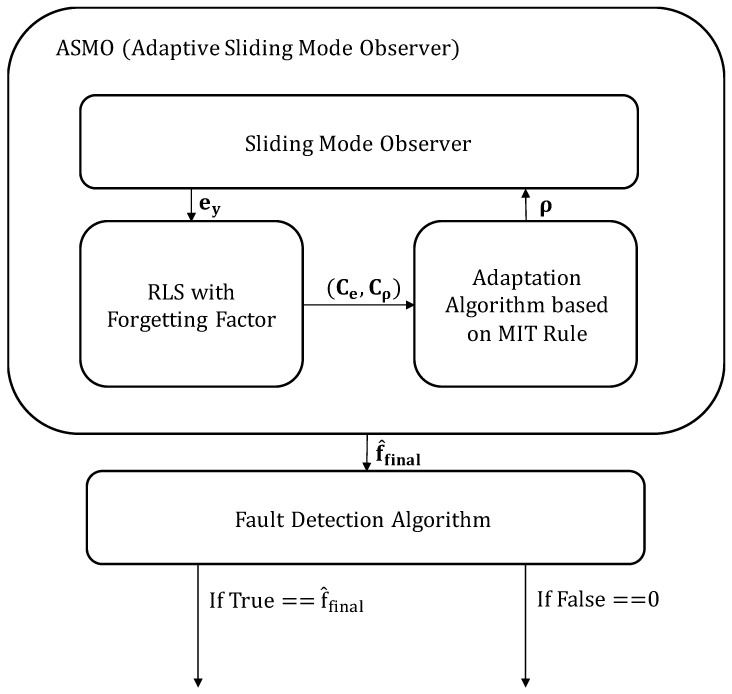
Model schematics of steering actuator bias detection and active tolerant-control.

Figure 2 schematically depicts the detailed procedure for actuator bias detection and AFTC. The fault-monitoring system uses a nominal linear vehicle model as a reference predictor. A residual is generated by comparing the measured vehicle states (from the nonlinear plant) with those predicted by the linear model. However, the residual contains both the effects of the actuator fault ($f_{a}$) and modeling uncertainties (d) because linear prediction cannot perfectly replicate nonlinear vehicle dynamics, particularly during dynamic maneuvers. The proposed ASMO is used to distinguish actual faults from such modeling errors. Unlike conventional observers, the proposed architecture integrates an adaptation algorithm based on the MIT rule WITH a recursive least squares (RLS) estimator utilizing a forgetting factor.

As illustrated in Figure 2, the observer reconstructs the unknown inputs, ensuring robust estimation even in the presence of model mismatches. Subsequently, a threshold-based fault detection algorithm monitors the estimated bias. Once the bias magnitude exceeds a predefined threshold (designed to accommodate nominal modeling errors), the fault is declared, and the reconstructed bias is subtracted from the steering command ($\delta_{cmd}$) to recover path tracking performance.

## 3. Lateral Vehicle Dynamics and Path Tracking Control

### 3.1. Vehicle Lateral Dynamics and Error Model

**Figure 3:**
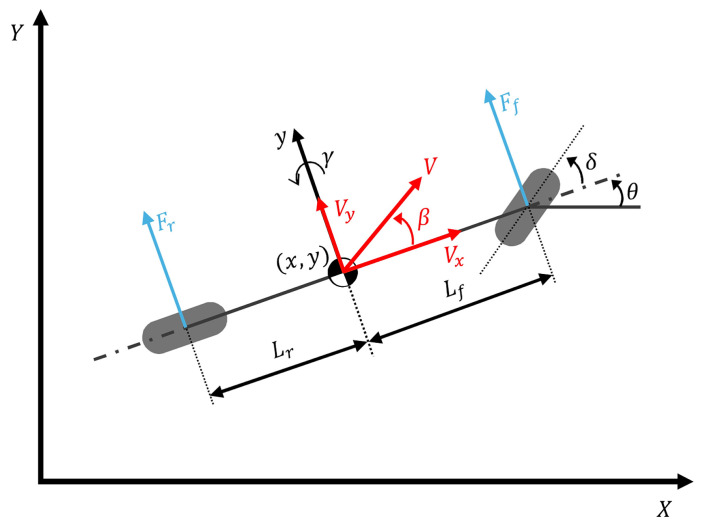
Lateral bicycle model of the vehicle for lateral dynamics.

To analyze the lateral motion of the vehicle for control purposes, a 2-DOF bicycle model is used, as shown in Figure 3. Assuming a planar road surface and small steering angles, the lateral dynamics are governed by Newton–Euler equations of motion applied to the center of gravity (CG) of the vehicle [21] as:

(2) $$ma_{y}=F_{yf}\left(\alpha_{f}\right)+F_{yr}(\alpha_{r}),$$

(3) $$I_{z}\dot{\gamma }=l_{f}F_{yf}\left(\alpha_{f}\right)-l_{r}F_{yr}(\alpha_{r}),$$

where m is the vehicle mass; $I_{z}$ is the yaw moment of inertia; and $l_{f}$ and $l_{r}$ denote the distances from the CG to the front and rear axles, respectively. The lateral acceleration $a_{y}$ consists of the lateral velocity derivative ${\dot{v}}_{y}$ and the centripetal term $v_{x}\gamma$ as:

(4) $$a_{y}={\dot{V}}_{y}+{\dot{V}}_{x}\gamma =V_{x}(\dot{\beta }+\gamma )$$

The lateral tire forces $F_{yf}$ and $F_{yr}$ represent the dominant factors that determine vehicle stability. In the linear region (small slip angles), these forces are proportional to the tire slip angles ($\alpha_{f},\;\alpha_{r}$) as:

(5) $$F_{yf}\approx 2C_{f}\left(\delta -\theta_{vf}\right),\;F_{yr}\approx -2C_{r}\theta_{vr},$$

where $C_{f}$ and $C_{r}$ denote the cornering stiffnesses of the front and rear tires, respectively. The slip angles are geometrically defined as [22]:

(6) $$\alpha_{f}=\delta -\frac{V_{y}-l_{r}\gamma }{V_{x}},\;\alpha_{r}=-\frac{V_{y}-l_{r}\gamma }{V_{x}},$$

However, in real-world driving scenarios involving high-speed cornering or low-friction roads, tire behavior is highly nonlinear. To accurately represent this reality in the simulation plant, the Pacejka Magic Formula is used as [23]:

(7) $$F_{y}=Dsin(C\;arctan\left(G\alpha -E\left(G\alpha -\arctan\left(G\alpha \right)\right)\right))$$

Since the control design is based on a nominal linear model (Equation (5)), the discrepancy between the actual nonlinear tire forces (Equation (7)) and the linear approximation is treated as a lumped uncertainty.

**Figure 4:**
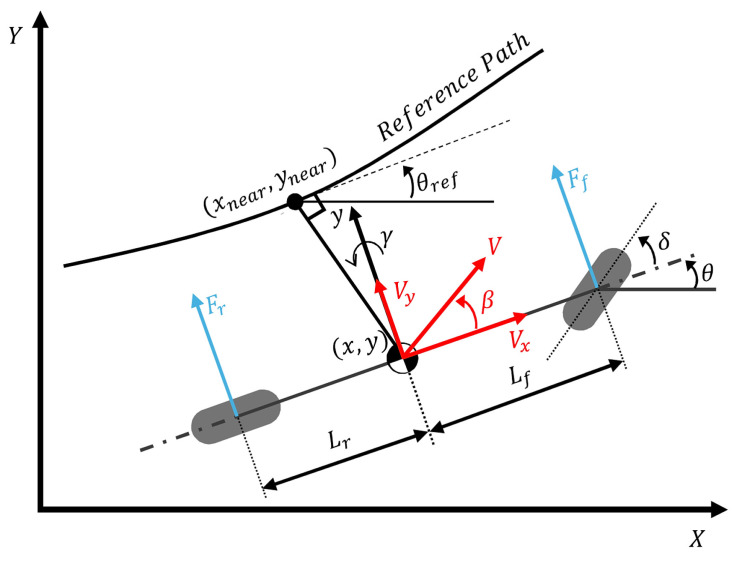
Path tracking error model.

For path tracking, the system is transformed into an error dynamics model relative to the reference path. The state vector is defined as $x={\left[\begin{array}{cccc} e_{y}, & {\dot{e}}_{y}, & e_{\psi }, & {\dot{e}}_{\psi } \end{array}\right]}^{T}$, where $e_{y}$ is the lateral distance error and $e_{\psi }$ is the heading angle error (see Figure 4) [24]. The resulting state-space equation, including the uncertainty term, can be expressed as:

(8) $$\dot{x}=Ax+Bu+d(x,u)$$

The system matrices A and B, derived from linearized dynamics, are given by:

(9) $$A=\left[\begin{array}{cccc} 0 & 1 & 0 & 0\\ 0 & -\frac{2C_{f}+2C_{r}}{mV_{x}} & \frac{2C_{f}+2C_{r}}{m} & -\frac{2C_{f}L_{f}-2C_{r}L_{r}}{mV_{x}}\\ 0 & 0 & 0 & 1\\ 0 & -\frac{2C_{f}L_{f}-2C_{r}L_{r}}{I_{z}V_{x}} & \frac{2C_{f}L_{f}-2C_{r}L_{r}}{I_{z}} & -\frac{2C_{f}L_{f}^{2}+2C_{r}L_{r}^{2}}{I_{z}V_{x}} \end{array}\right],B=\left[\begin{array}{c} 0\\ \frac{2C_{f}}{m}\\ 0\\ \frac{2C_{f}L_{f}}{I_{z}} \end{array}\right],$$

where the term d(x,u) aggregates the unmodeled dynamics, parameter variations, and external disturbances. This formulation provides a mathematical basis for the robust fault estimation scheme proposed in Section 4.

### 3.2. LQR Feedback Control

An LQR is designed to minimize tracking errors effectively. The algorithm calculates the optimal feedback gain K that minimizes the quadratic cost function [25] as:

(10) $$J=\int_{0}^{\infty }\left(x^{T}Qx+u^{T}Ru\right)dt,$$

where Q and R denote the weighting matrices. In this study, Q is a diagonal matrix $Q=diag(\begin{array}{cccc} q_{1}, & q_{2}, & q_{3}, & q_{4} \end{array})$ to prioritize specific error states while R represents the weight factor associated with the control input. The optimal control input can be expressed as:

(11) $$u_{fb}=-Kx=-R^{-1}B^{T}Px,$$

where P is the solution of the continuous-time algebraic Riccati equation (ARE).

(12) $$A^{T}P+PA-PBR^{-1}B^{T}P+Q=0$$

### 3.3. Curvature-Based Feedforward Control

**Figure 5:**
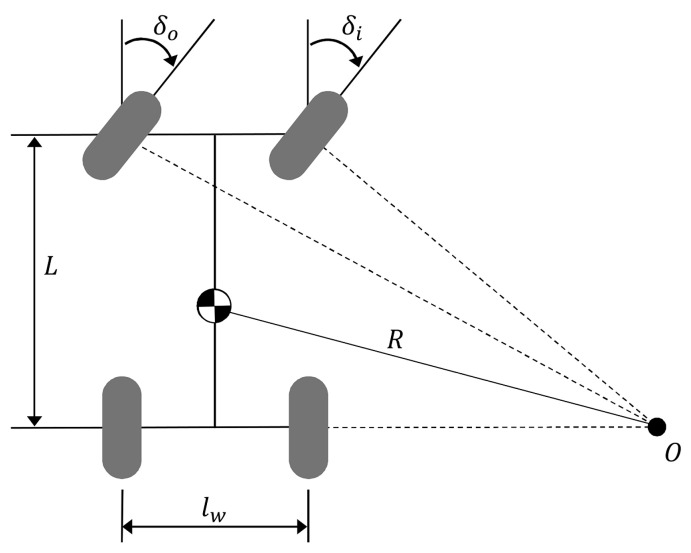
Geometry of a turning vehicle.

While the LQR controller handles state errors, a feedforward controller is introduced to improve the response time, particularly on curved roads. Based on the vehicle geometry shown in Figure 5, the steady-state steering angle required to negotiate a curve with radius $R_{path}$ is approximated using the bicycle model assumption as:

(13) $$\delta_{ff}\approx \frac{L}{R_{path}}=L\kappa_{ref},$$

where $L=l_{f}+l_{r}$ is the wheelbase and $\kappa_{ref}$ is the curvature of the reference path. The final steering command is the sum of the feedback and feedforward components [26]:

(14) $$\delta_{cmd}=u_{fb}+\delta_{ff}$$

## 4. Adaptive Actuator Fault Detection and Reconstruction, Active Tolerant Control Algorithm

### 4.1. Sliding Mode Observer for Fault Reconstruction of Steering Actuator Angle

Using the defined lateral state vector, the state-space equation of the vehicle in the presence of a steering actuator bias can be expressed as:

(15) $$\dot{x}=Ax+Bu+Ff_{b},$$

(16) y=Cx,

where $f_{b}$ denotes the bias fault input associated with the steering actuator. System matrices A and B are identical to those defined in Equation (9), whereas matrices F and C are expressed as:

(17) $$F=\left[\begin{array}{c} 0\\ 0\\ 0\\ -1 \end{array}\right],\;C=\left[\begin{array}{cccc} 1 & 1 & 1 & 1 \end{array}\right]$$

To design an SMO based on a state-space model that includes the steering actuator bias, an appropriate linear coordination transformation is first introduced to decouple the state variables from the output variables. For this purpose, the transformation matrix $T_{c}$ is defined as [27]:

(18) $$T_{c}=\left[\begin{array}{c} {Null(C)}^{T}\\ C \end{array}\right],$$

where NULL(C) denotes the null space of the output matrix C. By applying this transformation, the original state vector x can be represented in the new coordinate system as $x_{c}=T_{c}x$, and the transformed system dynamics can be expressed as:

(19) $${\dot{x}}_{c}=A_{c}x_{c}+B_{c}u+F_{c}f_{b},$$

(20) $$y=C_{c}x_{c},$$

where the transformed system matrices are given by:

(21) $$A_{c}=T_{c}AT_{c}^{-1},\;B_{c}=T_{c}B,\;C_{c}=CT_{c}^{-1},\;F_{c}=T_{c}F$$

This coordinate transformation separates the output-related states from the internal states, thereby simplifying the definition of the sliding surface and design of the injection term in the observer. Based on the transformed model, the observer is designed by incorporating an additional injection term (ν) as:

(22) $${\dot{\hat{x}}}_{c}=A_{c}{\hat{x}}_{c}+B_{c}u+G_{n}\nu ,$$

where ν denotes the observer injection term introduced to induce sliding mode behavior, and $G_{n}$ represents the corresponding distribution matrix defined as:

(23) $$G_{n}={\left[\begin{array}{cccc} L_{s1} & L_{s2} & L_{s3} & -1 \end{array}\right]}^{T}$$

$G_{n}$ contains the injection gains associated with each state error component and is designed to enable the rapid estimation of external disturbances, including steering actuator bias. Using this matrix, the observer maintains a stable sliding motion even in the presence of an actuator bias, and the reconstructed bias signal is subsequently utilized for steering input compensation. The elements $L_{s1},L_{s2},L_{s3}$ in the observer injection path $G_{n}$, defined in Equation (23), represent the design gains selected to ensure stable generation of the bias estimation signal. The injection term is defined using the sign function of the output error $e_{y}$ as:

(24) $$\nu =\rho sign(e_{y}),$$

where ρ>0 denotes the boundary gain that determines the reaching condition of the sliding surface. Defining the observer state estimation error as $e_{c}={\hat{x}}_{c}-x_{c}$, where ${\hat{x}}_{c}$ and $x_{c}$ denote the estimated and actual states in the transformed coordinates, respectively, the error dynamics can be expressed as:

(25) $${\dot{e}}_{c}=A_{c}e_{c}+G_{n}\nu$$

By decomposing Equation (25) into its individual components, the dynamics of each state-estimation error can be derived as:

(26) $${\dot{e}}_{1}=A_{c11}e_{1}+A_{c12}e_{2}+A_{c13}e_{3}+A_{c14}e_{y}+L_{s1}\nu$$

(27) $${\dot{e}}_{2}=A_{c21}e_{1}+A_{c22}e_{2}+A_{c23}e_{3}+A_{c24}e_{y}+L_{s2}\nu$$

(28) $${\dot{e}}_{3}=A_{c31}e_{1}+A_{c32}e_{2}+A_{c33}e_{3}+A_{c34}e_{y}+L_{s3}\nu$$

(29) $${\dot{e}}_{y}=A_{c41}e_{1}+A_{c42}e_{2}+A_{c43}e_{3}+A_{c44}e_{y}-\nu$$

To ensure the stable operation of the observer, the output estimation error $e_{y}$ must converge to zero within a finite time. Accordingly, the Lyapunov candidate function is used as [28]:

(30) $$V=\frac{1}{2}{\left(e_{y}\right)}^{2}$$

To guarantee the existence of sliding motion, the reaching condition $\dot{V}\leq -\alpha V^{\frac{1}{2}}(\alpha >0)$ is imposed, under which V(t) decreases to zero within a finite time. Consequently, the reaching time $t_{r}$ is bounded as:

(31) $$t_{r}\leq \frac{2V^{\frac{1}{2}}(0)}{\alpha }$$

To satisfy the stated condition, the injection gain ρ must be designed to dominate the interaction terms included in the output error dynamics. Therefore, the minimum gain condition can be expressed as [29]:

(32) $$\rho =L_{b}+\frac{\alpha }{\sqrt{2}},$$

where $L_{b}$ denotes the upper bound of the uncertainty and disturbance terms of the system. Based on the designed gains, the observer injection term can be rewritten by incorporating the coupling effects of the state estimation errors as:

(33) $$\nu =A_{c41}e_{1}+A_{c42}e_{2}+A_{c43}e_{3}$$

By substituting this expression into the error dynamics, the overall error system can be represented in the form of a multivariable feedback structure as:

(34) $$\dot{e}=\tilde{A}e+L_{s}\tilde{B}E,$$

where

(35) $$e={\left[\begin{array}{ccc} e_{1} & e_{2} & e_{3} \end{array}\right]}^{T},\tilde{A}=\left[\begin{array}{ccc} A_{c11} & A_{c12} & A_{c13}\\ A_{c21} & A_{c22} & A_{c23}\\ A_{c31} & A_{c32} & A_{c33} \end{array}\right],\;L_{s}=\left[\begin{array}{c} L_{s1}\\ L_{s2}\\ L_{s3} \end{array}\right],\;\tilde{B}=\left[\begin{array}{ccc} A_{c41} & A_{c42} & A_{c3} \end{array}\right]$$

Accordingly, the resulting multivariable interaction structure of the error dynamics can be generally summarized as:

(36) $${\dot{e}}_{i}=\sum_{j=1}^{3}(A_{cij}+L_{sj}A_{c4j})e_{j},\;i=1,2,3$$

Using the proposed model, interactions among the system states can be quantitatively characterized, enabling higher stability compared with conventional approaches based on signal-error dynamics. Once the state estimation errors converge to zero, the observer can reconstruct the steering actuator bias ${\hat{f}}_{b}$. Before applying this estimate for compensation, a fault-detection process is conducted to distinguish actual faults from modeling uncertainties or noise.

First, an instantaneous fault detection flag, denoted as $S_{raw}(k)$, is determined by comparing the magnitude of the reconstructed bias with a predefined error threshold $\epsilon_{fd}$ as:

(37) $$S_{raw}(k)=\{\begin{array}{c} 1,\;\left|{\hat{f}}_{b}(k)\right|\geq \epsilon_{d}\\ 0,\;\left|{\hat{f}}_{b}(k)\right|<\epsilon_{d}\; \end{array},$$

where $S_{raw}\left(k\right)=1$ indicates the potential fault condition. However, relying solely on this raw flag can lead to chattering owing to signal noise. Therefore, a robust validation logic is applied, as discussed further.

### 4.2. Gain Adaptive Strategy for Sliding Mode Observer

To ensure stable operation of the observer in the presence of a steering actuator bias, the sliding-mode observer incorporates an injection term that drives the output error to the sliding surface within a finite time. However, in practical vehicle applications, the uncertainty bounds of the system are neither constant nor known a priori due to modeling inaccuracies, variations in road conditions, and changes in tire cornering stiffness. Consequently, a fixed injection gain is insufficient to guarantee robustness under diverse driving conditions. Particularly, the appropriate magnitude of injection gain varies depending on the bias amplitude and the dynamic state of the vehicle, motivating the use of an adaptive gain strategy. To this end, the relationship between the injection gain ρ and the output error $e_{y}$ can be defined as:

(38) $$\left|e_{y}\right|=c_{e}\rho ,$$

(39) $$\left|\rho -\rho_{min}\right|=c_{\rho }\rho ,$$

where $c_{e}$ and $c_{\rho }$ denote adaptive coefficients that vary according to the system state. These coefficients can be estimated online using an RLS algorithm [30,31]. The linear regression model used for parameter estimation can be defined in terms of the output (y), the regression vector (ϕ), and the parameter vector (θ) as:

(40) $$y=\phi^{T}\theta$$

Furthermore, RLS gain ($R_{RLS}$), covariance matrix (P), and forgetting factor (λ) are used to update parameter estimation ($\hat{\theta }$) as:

(41) $$R_{RLS}=P\phi {\left(\lambda +\phi^{T}P\phi \right)}^{-1}$$

(42) $$\hat{\theta }=\left(\theta^{-}+R_{RLS}\left(y-\phi^{T}\theta^{-}\right)\right)$$

(43) $$P^{+}=(I-R_{RLS}\phi^{T})P^{-}\lambda^{-1}$$

The coefficients ($c_{e}$ and $c_{p}$) updated using the RLS scheme, are subsequently used to dynamically adjust the magnitude of the injection gain in the SMO. To formulate the adaptive law for injection gain, objective functions are defined as:

(44) $$V_{1}=\frac{1}{2}e_{y}^{2}$$

(45) $$V_{2}=\frac{1}{2}{\left(\rho -\rho_{min}\right)}^{2}$$

Objective function $V_{1}$ increases the injection gain when the output error exceeds a predefined boundary, whereas $V_{2}$ prevents excessive amplification of the injection gain when the output error remains within the nominal range. Applying the gradient descent method to these objective functions yields the adaptive update law, expressed as:

(46) $$\frac{d\rho_{1}}{dt}=-\gamma_{1}\frac{\partial V_{1}}{\partial \rho_{1}}=-\gamma_{1}e_{y}\frac{\partial e_{y}}{\partial \rho_{1}}=-\gamma_{1}e_{y}{\hat{c}}_{e}\;(\gamma_{1}>0)$$

(47) $$\frac{d\rho_{2}}{dt}=-\gamma_{2}\frac{\partial V_{2}}{\partial \rho_{2}}=-\gamma_{2}e_{\rho }\frac{\partial e_{\rho }}{\partial \rho_{2}}=-\gamma_{2}e_{\rho }{\hat{c}}_{\rho }\;(\gamma_{2}>0)$$

To selectively apply the appropriate objective function depending on the magnitude of output error, a threshold ($\varepsilon_{i}$), is introduced along with the switching conditions as:

(48) $$\left|e_{y}\right|\geq \varepsilon_{i},\;w_{e}=1\;\&\;w_{\rho }=0$$

(49) $$\left|e_{y}\right|<\varepsilon_{i},\;w_{e}=0\;\&\;w_{\rho }=1$$

Based on these switching rules, the adaptive injection gain can be expressed as:

(50) $$\rho =\int_{0}^{t}\left(-w_{e}\gamma_{1}e_{y}{\hat{c}}_{e}-w_{\rho }\gamma_{2}e_{\rho }{\hat{c}}_{\rho }\right)dt$$

The adaptive gain structure enables real-time adjustment of the SMO injection gain in response to variations in vehicle operating conditions and fault magnitude. Consequently, fast bias estimation and stable reconstruction performance can be achieved even in the presence of steering actuator bias faults. To establish a rigorous theoretical foundation for the proposed adaptive gain mechanism, Theorem 1 provides a Lyapunov-based stability analysis of the MIT rule and the RLS-integrated SMO.

**Theorem** **1.** *Boundedness and Reachability of the Adaptive Sliding Mode Observer.*

Consider the adaptive SMO defined by the error dynamics

$${\dot{e}}_{y}=A_{obs}e_{y}-\rho sign\left(e_{y}\right)+∆(t),$$

where $e_{y}$ denotes the output estimation error, ρ represents the adaptive injection gain updated according to the MIT rule, and Δ(t) denotes lumped uncertainties, including modeling errors and steering actuator bias effects.

Assume that lumped uncertainty is bounded such that $\left|∆(t)\right|\leq \bar{∆}$, where $\bar{∆}$ is a positive constant. If the adaptive injection gain satisfies $\rho >\bar{∆}$, then the output estimation error $e_{y}$ and the adaptive gain ρ are uniformly ultimately bounded. Furthermore, the estimation error $e_{y}$ converges to a neighborhood of the sliding surface in finite time.

**Proof of Theorem** **1.** To analyze the stability of the ASMO, consider the following Lyapunov function candidate:

$$V=\frac{1}{2}e_{y}^{2}+\frac{1}{2\gamma }{\left(\rho -\rho^{*}\right)}^{2},$$

where $\rho^{*}>\bar{∆}$ denotes an ideal injection gain and γ>0 is the adaptation rate.

The time derivative of V along the error dynamics is
  $$\dot{V}=e_{y}{\dot{e}}_{y}+\frac{1}{\gamma }(\rho -\rho^{*})\dot{\rho }$$
  Substituting the observer error dynamics into the above expression yields
  $$\dot{V}=e_{y}\left(A_{obs}e_{y}-\rho sign\left(e_{y}\right)+∆\left(t\right)\right)+\frac{1}{\gamma }(\rho -\rho^{*})\dot{\rho }$$
  The observer dynamics matrix $A_{obs}$ is designed to stable; therefore, the quadratic term $e_{y}A_{obs}e_{y}$ is non-positive.
The adaptive law derived from the MIT rule is designed to regulate the injection gain in response to the output estimation error, ensuring that the cross term involving $(\rho -\rho^{*})\dot{\rho }$ does not introduce instability.
Thus, the derivative of the Lyapunov function can be upper bounded as
  $$\dot{V}\leq -(\rho -\left|∆(t)\right|)\left|e_{y}\right|.$$
  Under the condition $\rho >\bar{∆}$, the inequality reduces to
  $$\dot{V}<0\;for\;e_{y}\neq 0,$$
  which guarantees the reachability condition $e_{y}{\dot{e}}_{y}<0$.
Consequently, the output estimation error $e_{y}$ converges to a neighborhood of the sliding surface within finite time.
The adaptive gain update mechanism further incorporates objective functions that prevent excessive gain amplification during nominal operation. In addition, the RLS-based parameter estimation ensures bounded adaptive coefficients under bounded regression signals. As a result, the adaptive injection gain ρ is uniformly ultimately bounded. □

### 4.3. Actuator Bias Fault Detection

The fault signal (${\hat{f}}_{a}$), estimated by the observer, exhibits high-frequency oscillations and zero-crossing phenomena, particularly during highly dynamic maneuvers such as a double lane change (DLC). As the steering input and lateral dynamics fluctuate rapidly, the estimated fault value momentarily passes through zero even when a valid fault exists.

Consequently, applying a simple static-thresholding method poses a significant risk, thereby causing the fault-detection flag to rapidly toggle between zero and one (chattering). This intermittent detection leads to discontinuous compensation signals, which may destabilize the vehicle control system. To mitigate this problem, a robust detection algorithm combining the root mean square (RMS) and off-delay logic is proposed.

To evaluate the intensity of the fault signal, regardless of its instantaneous zero-crossing direction, a moving RMS filter is applied. The RMS value represents the energy of the signal over a sliding window, providing a smoothed metric that remains positive even when the raw signal oscillates to zero. The moving RMS at the time step k can be defined as:

(51) $$RMS(k)=\sqrt{\frac{1}{N_{win}}\sum_{i=0}^{N_{win}-1}{\hat{f}}_{a}{\left(k-i\right)}^{2}},$$

where ${\hat{f}}_{a}(k)$ is the raw estimated fault signal and $N_{win}$ is the window size (number of samples). Given the system sampling time $T_{s}=0.01\;s$, the window size was set to $N_{win}=15$ (equivalent to a 0.15 s time window) for this study. The value is empirically selected to filter out zero-crossing noise sufficiently while maintaining a fast detection response time.

To further ensure robustness against transient signal drops and prevent the detection flag from flickering, a time-based hysteresis logic (off-delay timer) is used. The final fault detection status ($S_{fault}(k)$) is determined by monitoring the RMS value against a detection threshold ($\delta_{th}$) and recording the remaining time of the debouncing timer ($t_{timer}$). The logic can be mathematically defined as:

(52) $$S_{fault}(k)=\{\begin{array}{c} 1,\;if\;RMS(k)\geq \delta_{th}\\ 1,\;if\;RMS\left(k\right)<\delta_{th}\;and\;t_{timer}\left(k\right)>0\\ 0,\;otherwise \end{array}$$

If the RMS value exceeds the threshold, the timer is reset to the initial delay time ($T_{delay}$). Conversely, if the signal drops below the threshold but the timer remains active, the system maintains the fault status and decrements the timer $\left(t_{timer}\left(k+1\right)=t_{timer}\left(k\right)-T_{s}\right)$ until it reaches zero. Based on the validated fault status ($S_{fault}$), the final estimated fault signal (${\hat{f}}_{final}(k)$) used for compensation can be determined as:

(53) $${\hat{f}}_{final}(k)=\{\begin{array}{c} {\hat{f}}_{b}\left(k\right),\;if\;S_{fault}\left(k\right)=1\\ 0,\;if\;S_{fault}\left(k\right)=0 \end{array}$$

Finally, AFTC can be achieved by subtracting the validated bias estimate from the steering command. The actual steering angle ($\delta_{actual}$) is effectively compensated for the bias as:

(54) $$\delta_{actual}=\left(\delta_{cmd}-f_{b}\right)-{\hat{f}}_{final}\approx \delta_{cmd}$$

This structure ensures that compensation is activated only when the fault is confidently validated, effectively preventing unintended noise or residual estimation errors from destabilizing the lateral dynamics of the vehicle during normal operation. The detection and persistence thresholds are selected to exceed the RMS variations observed under fault-free conditions, while remaining sufficiently sensitive to ensure timely fault declaration in the presence of a persistent actuator bias. This selection achieves a reliable trade-off between false-alarm suppression and detection responsiveness.

## 5. Performance Evaluation and Analysis

To evaluate the effectiveness of the proposed steering actuator bias detection and AFTC algorithm, a Simulink-based simulation environment was developed to reproduce autonomous vehicle path-tracking behavior under fault conditions. The overall system architecture integrated polynomial-based path generation, curvature-based feedforward steering, LQR-based feedback control, and a 3-DOF dual-track vehicle model to realistically capture the lateral and longitudinal vehicle dynamics. The steering actuator bias faults were artificially injected into the steering command, and the system performance was evaluated over a vehicle speed range of 30–100 km/h under three conditions: (i) normal driving without faults, (ii) bias injection without compensation, and (iii) bias compensation using the proposed ASMO-based algorithm. The performance metrics included vehicle trajectory, lateral distance error, and heading error, which were quantitatively assessed using RMS values.

Although the observer is designed based on a linearized vehicle model, the simulation environment incorporates nonlinear tire dynamics to evaluate realistic high-speed driving conditions. The resulting model-plant mismatch is intentionally treated as structured uncertainty to rigorously evaluate the robustness of the proposed algorithm. To comprehensively assess the technical contribution, the performance of the proposed ASMO is compared not only with a conventional fixed-gain SMO within the same observer family but also with mainstream filtering-based methods, specifically the extended Kalman filter (EKF) and standard Kalman Filter (KF). This comparative analysis demonstrates the enhanced robustness of the adaptive sliding mode approach under nonlinear model uncertainties and time-varying faults, when compared with conventional recursive estimation techniques.

To this end, three representative bias scenarios are considered: a sudden (step) bias, a gradually increasing drift bias, and a constant initial bias. These scenarios evaluate transient response, steady-state accuracy, and stability under varied fault characteristics.

A single set of observer and adaptive gain parameters is applied consistently across all scenarios, ensuring that the results reflect the inherent robustness of the proposed system.

Among the tested conditions, the results at 100 km/h are presented as representative cases since high-speed driving introduces stronger lateral dynamics and tighter stability margins, thereby imposing more stringent requirements on fault detection and compensation performance. The vehicle specifications used in the simulations are listed in Table 1 and are derived from real-world vehicle data to ensure authenticity.

### 5.1. Constant Steering Actuator Bias Reconstruction on a DLC Path (100 km/h)

**Figure 6:**
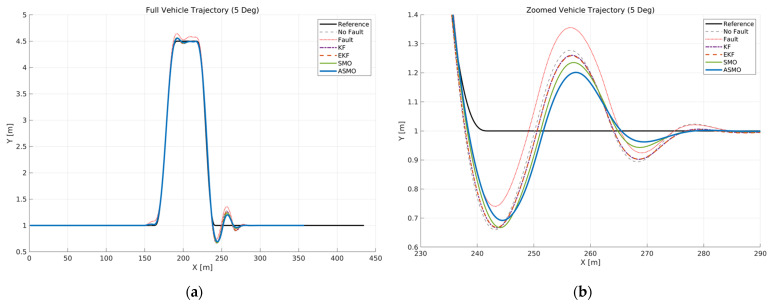
Vehicle trajectory for Scenario 1: Comparison of reference path, nominal case, steering bias fault (5 deg), and active fault-tolerant controls (ASMO, SMO, EKF, KF): (**a**) Overall vehicle trajectory comparison under Scenario 1 (5 deg steering actuator bias); (**b**) Magnified view of the critical curvature transition region highlighting tracking deviations among fault reconstruction methods.

Figure 6 illustrates the vehicle trajectory tracking performance under Scenario 1, where a constant 5 deg steering actuator bias was applied at a longitudinal speed of 100 km/h. The reference trajectory is compared with the uncompensated faulty case as well as KF, EKF, SMO, and ASMO-based compensation methods.

As observed in the global view (Figure 6a), the uncompensated case exhibits significant lateral deviation and oscillatory behavior throughout the DLC maneuver due to the persistent steering bias. The KF- and EKF-based approaches reduce the overall tracking error compared to the uncompensated case; however, noticeable deviations remain near the peak lateral distance and during the returning phase.

The enlarged view (Figure 6b) further reveals the transient tracking characteristics around the curvature transition region. While the KF and EKF partially attenuate the bias effect, residual tracking errors are still evident. The SMO-based method improves stability compared to KF and EKF, yet small deviations persist. In contrast, the proposed ASMO-based FTC maintains close alignment with the reference trajectory, effectively compensating for the steering bias and achieving tracking performance comparable to the nominal fault-free condition.

**Figure 7:**
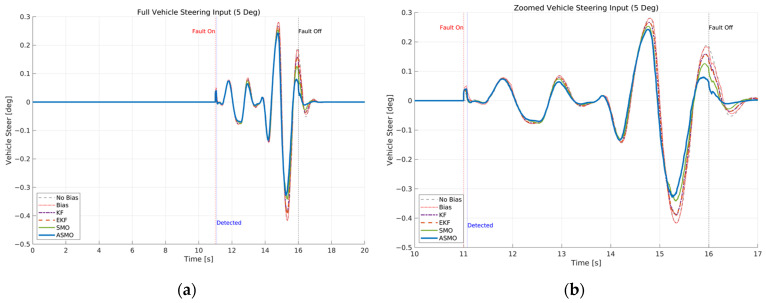
Comparison of vehicle input steering profiles in Scenario 1: nominal case, steering bias fault (5 deg), and active fault-tolerant control strategies (ASMO, SMO, EKF, KF): (**a**) Overall steering input profiles for nominal, bias, and fault-tolerant control methods; (**b**) Enlarged view of the post-fault transient region highlighting differences in peak steering correction and oscillation behavior.

**Figure 8:**
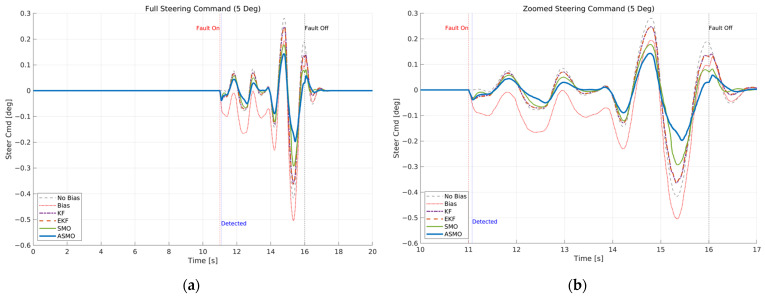
Comparison of controller steering commands in Scenario 1 under different conditions: nominal, steering bias fault (5 deg), and active fault-tolerant controls (ASMO, SMO, EKF, KF): (**a**) Overall steering command responses for nominal, bias, and fault-tolerant control methods; (**b**) Enlarged view of the post-fault transient region highlighting differences in peak compensation effort and oscillatory behavior.

Figure 7 and Figure 8 illustrate the steering interaction between the controller and the vehicle dynamics in the presence of a 5 deg steering actuator bias. Figure 8 presents the steering command generated by each control strategy, whereas Figure 7 shows the actual steering input applied to the vehicle after the bias is injected.

In the uncompensated case, the steering command remains nearly identical to the nominal condition because the controller is unaware of the actuator fault. As a result, the actual steering input is shifted by the bias magnitude, leading to a persistent offset in the vehicle response. The KF- and EKF-based approaches partially adjust the steering command to counteract the bias; however, residual overcompensation and transient oscillations remain observable. The SMO-based strategy further improves the compensation performance, yet slight deviations in peak steering response are still present. In contrast, the proposed ASMO-based controller actively modifies the steering command in the direction opposite to the fault, as illustrated in Figure 8. Consequently, the actual steering input in Figure 7 closely aligns with the nominal “No Bias” profile, effectively mitigating the 5 deg actuator offset.

**Figure 9:**
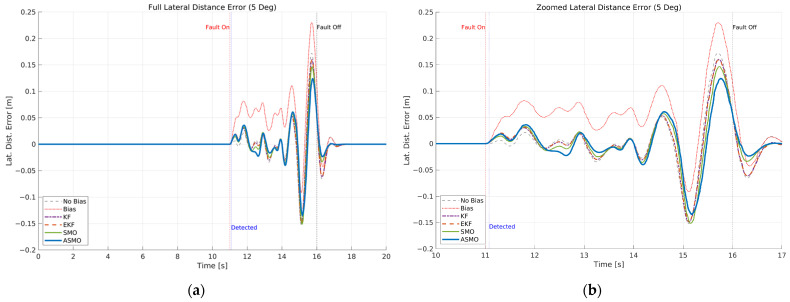
Evaluation of lateral distance error in Scenario 1: Comparison between the nominal case, steering bias fault (5 deg), and active fault-tolerant controls (ASMO, SMO, EKF, KF): (**a**) Overall lateral error responses for nominal, bias, and fault-tolerant control methods; (**b**) Enlarged view of the post-fault transient region highlighting differences in peak tracking error and oscillation characteristics.

**Figure 10:**
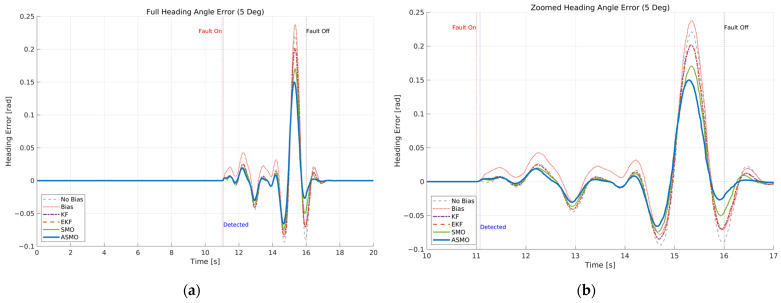
Evaluation of heading angle error in Scenario 1: Comparison between the nominal case, steering bias fault (5 deg), and active fault-tolerant controls (ASMO, SMO, EKF, KF): (**a**) Overall heading error responses for nominal, bias, and fault-tolerant control strategies; (**b**) Enlarged view of the post-fault transient region highlighting differences in peak orientation error and damping characteristics.

The resulting tracking performance is quantified in Figure 9 and Figure 10, where the lateral distance error and heading angle error are presented, respectively. The uncompensated vehicle exhibits substantial tracking deviations, particularly during the curvature transition and returning phases of the DLC maneuver. The KF and EKF methods reduce the magnitude of these errors compared to the uncompensated case, while the SMO approach provides further improvement in damping characteristics. Nevertheless, residual peak deviations remain visible. The ASMO-based control strategy significantly suppresses both lateral and heading errors, achieving tracking behavior nearly identical to the fault-free nominal condition. These results indicate that the proposed observer enables accurate bias estimation and enhances closed-loop fault-tolerant performance.

**Figure 11:**
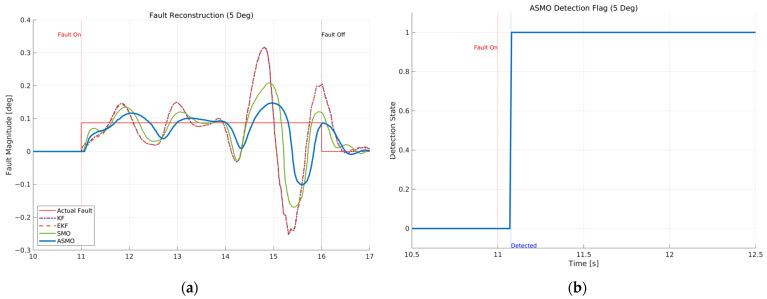
Fault reconstruction and detection performance under Scenario 1 (5 deg steering actuator bias): (**a**) Comparison between actual steering bias and reconstructed fault magnitudes using KF, EKF, SMO, and ASMO observers; (**b**) ASMO-based fault detection flag indicating rapid and stable fault identification after bias injection.

Figure 11a compares the actual bias magnitude with the reconstructed fault estimates obtained from KF-, EKF-, SMO-, and ASMO-based observers. The KF and EKF approaches exhibit noticeable estimation amplitude mismatch and phase lag, particularly during rapid steering transitions. The SMO method improves estimation accuracy and reduces phase delay; however, discrepancies remain in the peak regions. In contrast, the proposed ASMO demonstrates close agreement with the actual fault profile across the entire maneuver, indicating enhanced estimation precision and robustness under dynamic conditions.

Figure 11b illustrates the corresponding ASMO-based fault detection flag. The detection signal transitions promptly from 0 to 1 immediately after fault injection and remains stable without chattering or false alarms throughout the maneuver. This rapid and reliable detection ensures timely activation of the FTC mechanism, thereby contributing to the improved tracking performance observed in Figure 7, Figure 8, Figure 9 and Figure 10.

**Figure 12:**
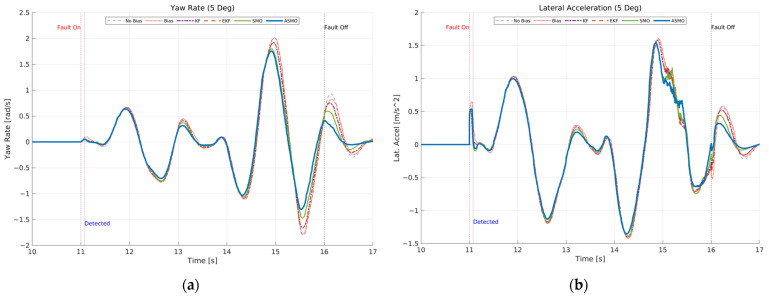
Transient vehicle dynamics response under Scenario 1 (5 deg steering actuator bias): (**a**) Yaw rate response comparison; (**b**) Lateral acceleration response comparison.

Figure 12 presents the transient vehicle dynamic responses under 5 deg steering actuator bias condition. Figure 12a illustrates the yaw rate response. In the uncompensated case, the persistent steering bias results in amplified yaw rate peaks and noticeable phase distortion during the curvature transition of the DLC maneuver. The KF- and EKF-based approaches reduce the overall deviation compared to the uncompensated case; however, residual peak amplification and slight oscillatory behavior remain. The SMO method further improves damping characteristics, yet small discrepancies are still observed near maximum yaw rate. In contrast, the proposed ASMO-based control maintains yaw rate dynamics closely aligned with the nominal fault-free response, indicating effective restoration of rotational stability. Figure 12b shows the corresponding lateral acceleration response. Similar trends are observed: the uncompensated bias leads to increased acceleration peaks and asymmetry between positive and negative responses, reflecting degraded lateral stability. The KF and EKF methods provide partial compensation, while the SMO approach further attenuates oscillations. The ASMO-based strategy achieves lateral acceleration behavior nearly identical to the nominal case, demonstrating robust dynamic stability despite the presence of actuator bias.

These results confirm that accurate fault reconstruction not only improves tracking performance but also preserves the intrinsic vehicle dynamic characteristics, ensuring stable transient behavior under actuator fault conditions.

**Figure 13:**
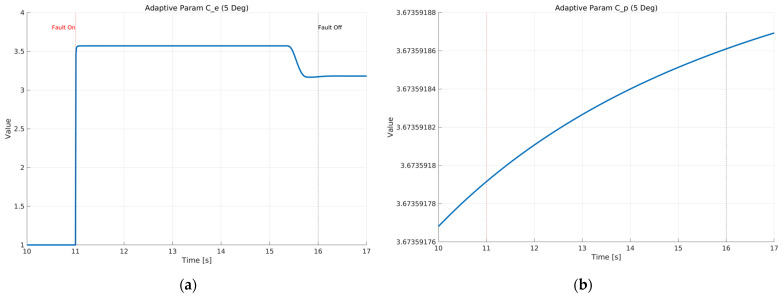
Time evolution of adaptive parameters in Scenario 1 (5 deg steering actuator bias): (**a**) Adaptive parameter $C_{e}$; (**b**) Adaptive parameter $C_{\rho }$.

Figure 13 shows the time histories of the adaptive parameters $C_{e}$ and $C_{\rho }$ in the proposed ASMO under Scenario 1 with a constant 5 deg steering actuator bias at 100 km/h. As depicted in Figure 13a, $C_{e}$ increases sharply at the fault injection instant, indicating an immediate gain adjustment triggered by the abrupt change in the effective steering input. After this rapid transition, $C_{e}$ remains bounded and nearly constant during the fault-active interval, and then slightly decreases when the fault is removed, suggesting a stable re-adaptation toward the nominal operating condition. Figure 13b presents the evolution of $C_{\rho }$, which changes gradually and monotonically after fault injection. The absence of chattering or divergent growth demonstrates that the adaptive law provides a well-damped gain update, maintaining parameter boundedness while supporting accurate fault reconstruction. This stable adaptation behavior is consistent with the improved tracking and error suppression results shown in the preceding figures.

**Figure 14:**
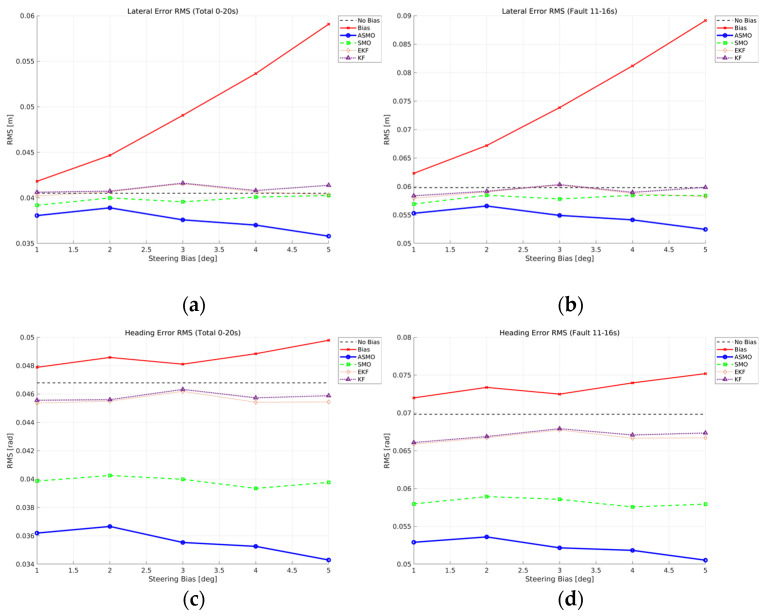
RMS tracking performance under varying steering actuator bias magnitudes (1–5 deg): (**a**) Lateral distance RMS over the entire maneuver (0–20 s); (**b**) Lateral distance RMS during the fault-active interval (11–16 s); (**c**) Heading angle RMS over the entire maneuver (0–20 s); (**d**) Heading angle RMS during the fault-active interval (11–16 s).

Figure 14a,c present the RMS values of lateral distance and heading angle errors over the entire maneuver (0–20 s). In the uncompensated case, both lateral and heading RMS errors increase monotonically with bias magnitude, indicating progressive degradation of tracking performance. The KF and EKF methods reduce the overall error compared to the uncompensated case; however, their RMS levels remain relatively insensitive to increasing bias, reflecting limited compensation capability. The SMO approach further improves performance by maintaining lower RMS values across all bias levels.

The superiority of the proposed ASMO becomes more pronounced when focusing on the fault-active interval (11–16 s), as shown in Figure 14b,d. While the uncompensated case exhibits a clear bias-dependent growth in RMS error, the ASMO-based control maintains the lowest error levels and demonstrates strong robustness against increasing fault magnitude. Notably, the RMS variation with respect to bias amplitude remains minimal, indicating effective bias reconstruction and consistent fault-tolerant performance.

Table 2 summarizes the quantitative performance indices of fault detection and estimation under Scenario 1 for steering bias magnitudes ranging from 1 to 5 deg. First, the fault detection delay decreases consistently as the bias magnitude increases. While a 1 deg bias requires approximately 606 ms for detection, the delay reduces to approximately 76 ms at 5 deg. This trend indicates that larger fault magnitudes produce more distinguishable residual dynamics, enabling faster fault identification.

Importantly, the false alarm rate (FAR) and missed detection rate (MDR) remain zero across all tested bias levels. This confirms the reliability and robustness of the proposed detection scheme, demonstrating stable discrimination between normal and faulty conditions without chattering or misclassification. The improvement indices for lateral distance error and heading angle error further validate the effectiveness of the FTC. As the bias magnitude increases, the improvement ratios also increase, reaching over 40% in lateral error reduction and approximately 33% in heading error reduction at 5 deg. This indicates that the proposed method becomes increasingly beneficial under more severe actuator faults.

Overall the results demonstrate that the ASMO-based framework achieves rapid and reliable fault detection while providing substantial tracking performance recovery, particularly under larger bias conditions.

### 5.2. Intermittent Steering Actuator Bias Reconstruction on a DLC Path (100 km/h)

**Figure 15:**
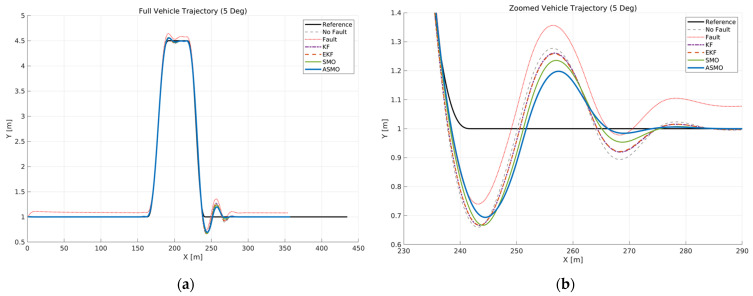
Vehicle trajectory for Scenario 2: Comparison of reference path, nominal case, steering bias fault (5 deg), and active fault-tolerant controls (ASMO, SMO, EKF, KF): (**a**) Overall trajectory during the double-lane-change maneuver; (**b**) Enlarged view of the curvature transition region highlighting tracking deviation among compensation methods.

Figure 15 presents the vehicle trajectory under Scenario 2, where an intermittent 5 deg steering actuator bias is introduced at 100 km/h. This scenario evaluates the transient behavior of the AFTC system when the fault appears and disappears abruptly during the DLC maneuver. As shown in the enlarged view, the uncompensated case exhibits noticeable lateral distance immediately after fault activation, resulting in trajectory distortion during the curvature transition phase. When the fault is removed, additional transient deviation is observed due to the abrupt change in effective steering input. The KF- and EKF-based approaches reduce the magnitude of these deviations; however, residual tracking errors remain during both activation and recovery phases. The SMO-based method further attenuates the deviation and improves stability, yet small discrepancies persist near the peak lateral distance. In contrast, the proposed ASMO-based strategy maintains a trajectory closely aligned with the nominal “No Bias” case throughout the intermittent fault interval. The adaptive detection and compensation mechanism responds promptly to both fault onset and removal, effectively mitigating transient disturbances and preserving lateral stability during the maneuver. These results demonstrate the robustness of the proposed approach under rapidly varying actuator fault conditions.

**Figure 16:**
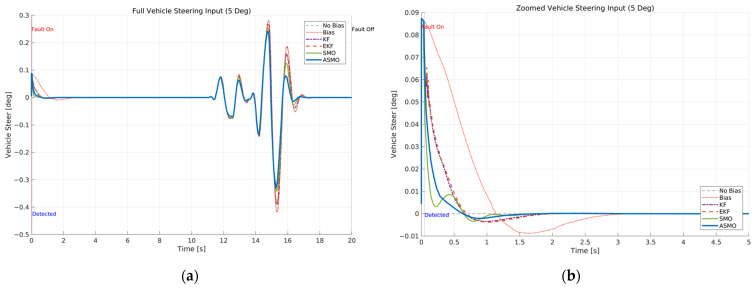
Vehicle steering input responses under Scenario 2: (**a**) Overall steering input during the double lane change maneuver; (**b**) Enlarged view of the transient response immediately after fault activation.

**Figure 17:**
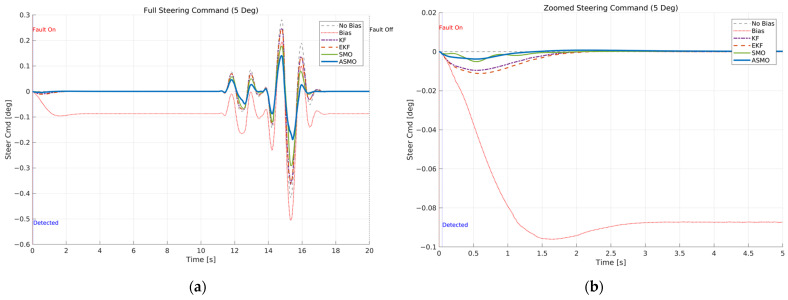
Controller steering command responses under Scenario 2: (**a**) Overall steering command during the double lane change maneuver; (**b**) Enlarged view of the transient control effort immediately after fault activation.

**Figure 18:**
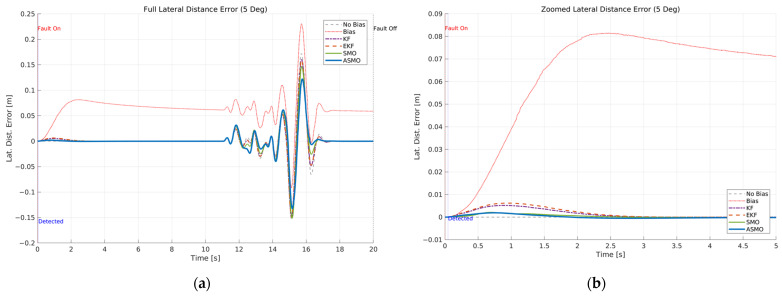
Lateral distance error under Scenario 2: (**a**) Overall lateral error during the double lane change maneuver; (**b**) Enlarged view of the transient response immediately after fault activation.

**Figure 19:**
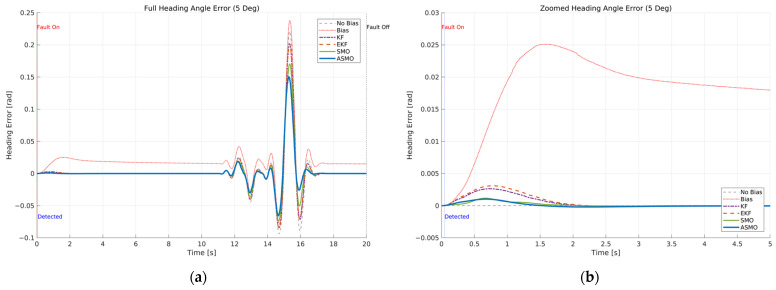
Heading angle error under Scenario 2: (**a**) Overall heading error response; (**b**) Enlarged transient response following fault activation.

Figure 16 and Figure 17 illustrate the internal operation of the AFTC mechanism under the intermittent steering actuator bias scenario. Figure 17 presents the steering command generated by each control strategy, while Figure 16 shows the corresponding actual steering input applied to the vehicle after the actuator bias is introduced. During the fault-active internal, the uncompensated controller generates a steering command nearly identical to that of the nominal case, as it does not account for the actuator bias. Consequently, the actual steering input exhibits a persistent offset proportional to the fault magnitude. The KF- and EKF-based methods partially modify the steering command to compensate for the bias; however, noticeable transient overcompensation and residual deviations remain during both fault activation and removal. The SMO-based controller further reduces these discrepancies but still shows minor peak deviations in the transient phases. In contrast, the proposed ASMO-based controller actively adjusts the steering command in the direction opposite to the bias during fault activation and re-adjusts it promptly upon fault removal. As a result, the actual steering input in Figure 16 closely follows the nominal “No Bias” profile throughout the intermittent fault interval, demonstrating effective mitigation of the 5 deg actuator offset. The impact of this compensation on tracking performance is quantified in Figure 18 and Figure 19, which present the lateral distance error and heading angle error, respectively. In the uncompensated case, the intermittent fault produces abrupt increases in both lateral and heading errors at the moments of fault onset and removal, reflecting degraded transient tracking behavior. The KF and EKF approaches reduce the magnitude of these deviations, while the SMO method provides further improvement in damping characteristics. Nevertheless, residual peak errors remain observable. The proposed ASMO-based control significantly suppresses both lateral and heading error peaks and enables faster convergence toward the nominal response after each fault transition. These results indicate that the adaptive gain mechanism of the ASMO enhances transient responsiveness and estimation precision compared to fixed-gain approaches, ensuring robust tracking performance under rapidly varying actuator fault conditions.

**Figure 20:**
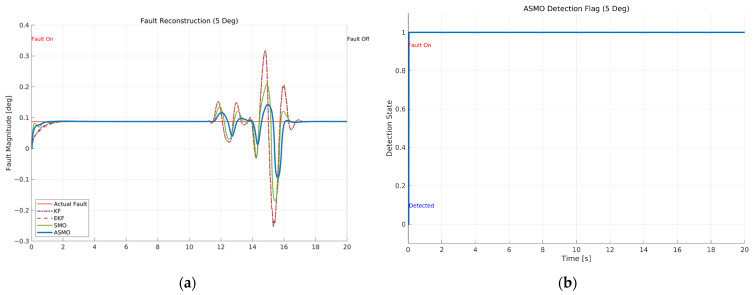
Fault reconstruction and detection performance under Scenario 2: (**a**) Comparison between actual steering bias and reconstructed fault magnitude using KF, EKF, SMO, and ASMO observers; (**b**) ASMO-based fault detection flag indicating rapid activation and stable fault identification.

Figure 20 presents the fault reconstruction and detection performance under the intermittent steering actuator bias scenario at 100 km/h. As shown in Figure 20a, the proposed ASMO-based estimator closely follows the injected bias profile throughout both the activation and removal intervals. Although small estimation fluctuations appear during the highly dynamic phase of the DLC maneuver (approximately 10–20 s), the reconstructed fault magnitude remains bounded and closely aligned with the actual bias. In comparison, the KF and EKF approaches exhibit noticeable amplitude mismatch and phase delay, while the SMO method shows larger peak deviations during rapid steering transitions. These results indicate that the adaptive mechanism in the ASMO improves estimation accuracy under rapidly varying dynamic conditions. Figure 20b illustrates the corresponding fault detection flag generated by the ASMO-based logic. The detection signal transitions promptly to the fault state immediately after bias activation and returns consistently upon fault removal, without exhibiting chattering behavior. The stable high-state maintenance during the fault-active interval demonstrates that the detection scheme effectively discriminates between normal dynamic variations and actual actuator faults. This reliable detection performance ensures timely engagement of the FTC mechanism under intermittent fault conditions.

**Figure 21:**
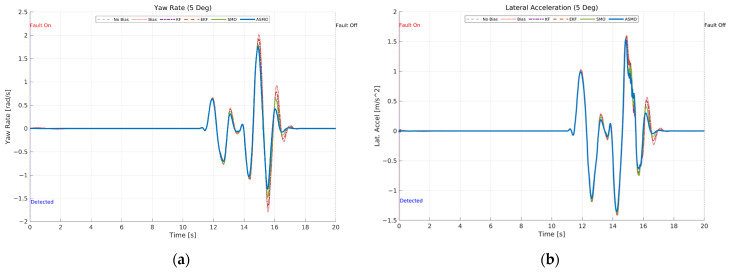
Transient vehicle dynamics response under Scenario 2: (**a**) Yaw rate response comparison; (**b**) Lateral acceleration response comparison.

Figure 21 presents the transient vehicle dynamic responses under Scenario 2 with an intermittent 5 deg steering actuator bias at 100 km/h. The yaw rate and lateral acceleration responses are shown to evaluate the impact of intermittent fault transitions on vehicle stability. In the uncompensated case, fault activation leads to amplified yaw rate peaks and asymmetric lateral acceleration responses, particularly during the high-curvature phase of the DLC maneuver. These deviations become more pronounced at the moments of fault activation and removal, reflecting the abrupt change in effective steering input.

The KF- and EKF-based methods reduce the magnitude of these deviations; however, residual peak amplification and light phase discrepancies remain observable. The SMO-based approach further improves damping characteristics, yet small differences from the nominal response persist during rapid transient phases. In contrast, the proposed ASMO-based control maintains yaw rate and lateral acceleration responses closely aligned with the fault-free nominal condition throughout both the fault-active and recovery intervals. The dynamic responses remain symmetric and well-damped, indicating that the adaptive compensation effectively preserves vehicle stability under intermittent fault switching conditions.

These results confirm that accurate fault reconstruction not only improves tracking accuracy but also maintains the intrinsic dynamic characteristics of the vehicle during rapid fault transitions.

**Figure 22:**
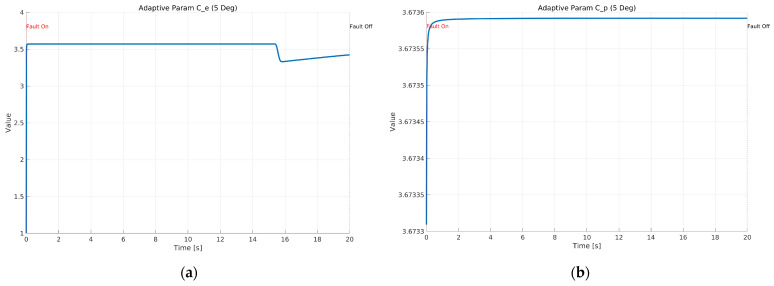
Time evolution of adaptive parameters under Scenario 2: (**a**) Adaptive parameter $C_{e}$ showing rapid gain adjustment during fault activation and re-adjustment after fault removal; (**b**) Adaptive parameter $C_{\rho }$ exhibiting bounded and stable behavior throughout the maneuver.

Figure 22 illustrates the self-tuning mechanism of the ASMO parameters in response to an intermittent fault. As shown in Figure 21a, the adaptive parameter $C_{e}$ undergoes a rapid adjustment at the moment of fault activation, reflecting an immediate increase in observer gain to enhance sensitivity to the abrupt change in system dynamics. When the fault is removed, $C_{e}$ readjusts smoothly toward its nominal level. Importantly, the parameter remains bounded throughout the maneuver, indicating stable adaptation without excessive gain amplification. Figure 21b presents the evolution of $C_{\rho }$, which converges rapidly during the initial phase and remains nearly constant during the fault-active interval. Unlike $C_{e}$, $C_{\rho }$ does not exhibit abrupt variations at fault transition, demonstrating that the adaptive mechanism selectively modifies observer gains according to their functional roles. This coordinated adaptation allows the observer to respond quickly to sudden dynamic change while maintaining robustness against oscillatory or chattering behavior. Overall, the bounded and well-structured parameter evolution confirms that the adaptive law provides fast reconfiguration capability under intermittent fault conditions, contributing to the improved transient tracking and stability performance observed in the preceding trajectory and error results.

**Figure 23:**
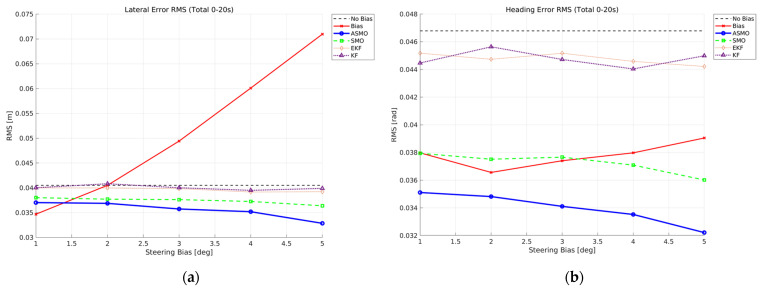
RMS tracking performance under Scenario 2: (**a**) Lateral distance RMS over the entire maneuver; (**b**) Heading angle RMS over the entire maneuver.

Figure 23 provides a quantitative comparison of tracking performance under intermittent steering actuator bias conditions in Scenario 2. The RMS values of lateral distance and heading angle errors are evaluated for fault magnitudes ranging from 1 to 5 deg. In the uncompensated case, the RMS errors increase noticeably as the fault magnitude grows, reflecting the cumulative impact of repeated fault activation and removal during the maneuver. This trend indicates that intermittent actuator bias can produce tracking degradation comparable to persistent faults if no compensation mechanism is applied. The KF and EKF methods reduce the overall RMS levels relative to the uncompensated case; however, their performance remains relatively insensitive to increasing fault magnitude, suggesting limited responsiveness to rapid fault transitions. The SMO-based controller further decreases the RMS values, demonstrating improved disturbance rejection. Nevertheless, residual error levels remain higher than those achieved by the proposed ASMO method. In contrast, the ASMO-based strategy consistently achieves the lowest RMS errors across all tested fault magnitudes. By adaptively adjusting observer gains during both fault activation and removal phases, the ASMO effectively suppresses transient peak deviations and accelerates convergence to the nominal trajectory. These results demonstrate that the adaptive mechanism enhances robustness against time-varying intermittent actuator faults, particularly under rapid switching conditions.

Table 3 summarizes the quantitative performance indices of fault detection and estimation under Scenario 2 with intermittent steering actuator bias ranging from 1 to 5 deg. First, the fault detection delay decreases consistently as the fault magnitude increases. A 1 deg intermittent bias requires approximately 167 ms for detection, while the delay reduces to approximately 47 ms at 5 deg. Similar to Scenario 1, larger fault magnitudes generate more distinguishable residual responses, enabling faster detection even under intermittent activation conditions. Importantly, the FAR and MDR remain zero across all tested magnitudes. This confirms that the proposed detection logic reliably discriminates between normal maneuver-induced dynamics and actual actuator faults, without chattering or misclassification during repeated fault activation and removal.

The improvement indices for lateral distance error and heading angle error further quantify the benefit of FTC. For small fault magnitudes (e.g., 1 deg), the improvement in lateral error is marginal and slightly negative, reflecting the limited impact of very small intermittent bias on overall RMS performance. However, as the fault magnitude increases, the improvement ratios grow substantially, reaching over 53% reduction in lateral error and approximately 17% reduction in heading error at 5 deg. This indicates that the proposed method becomes increasingly effective under more severe intermittent fault conditions.

Overall, the results demonstrate that the ASMO-based framework achieves rapid and reliable detection while maintaining robust tracking performance under time-varying intermittent actuator faults.

### 5.3. Drift-Type Steering Actuator Bias Reconstruction on a DLC Path (100 km/h)

**Figure 24:**
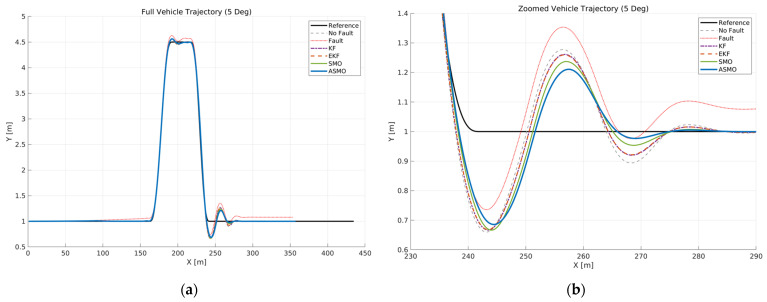
Vehicle trajectory comparison under Scenario 3: (**a**) Overall trajectory during the double lane change maneuver; (**b**) Enlarged view of the curvature transition region highlighting tracking deviation among compensation methods.

Figure 24 illustrates the vehicle trajectory under a drift-type steering actuator bias at 100 km/h. The left plot shows the overall DLC trajectory, while the right plot provides a magnified view of the curvature transition region. In the uncompensated case, the gradual accumulation of steering bias leads to progressive deviation from the reference path, particularly after the peak curvature phase of the maneuver. Unlike a constant bias fault, the drift-type fault introduces a time-varying offset that continuously alters the effective steering input, resulting in increasing lateral misalignment during the recovery phase.

The KF- and EKF-based approaches reduce part of this accumulated deviation; however, noticeable residual tracking errors remain due to limited adaptability to slowly varying fault dynamics. The SMO-based method further improves path tracking stability, yet visible discrepancies persist during the later stages of the maneuver, reflecting incomplete compensation of the drift component. In contrast, the proposed ASMO-based controller maintains a trajectory closely aligned with the nominal no-fault condition throughout the maneuver. By adaptively updating the observer gains in response to the time-varying bias, the ASMO effectively suppresses the accumulated deviation and restores convergence toward the reference lane. These results demonstrate that the proposed approach provides robust compensation against gradually evolving actuator faults while preserving lateral tracking stability.

**Figure 25:**
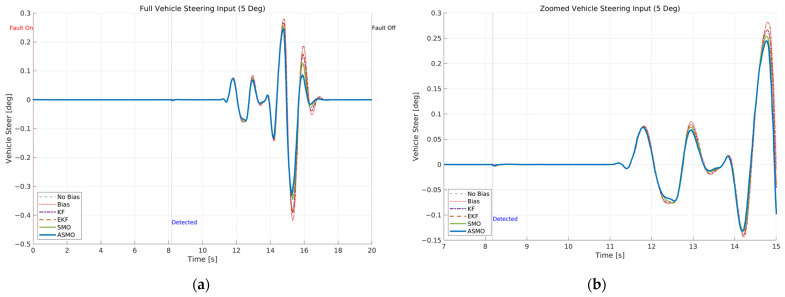
Vehicle steering input responses under Scenario 3: (**a**) Overall steering input during the double lane change maneuver; (**b**) Enlarged view of the transient steering response during the drift-active interval.

**Figure 26:**
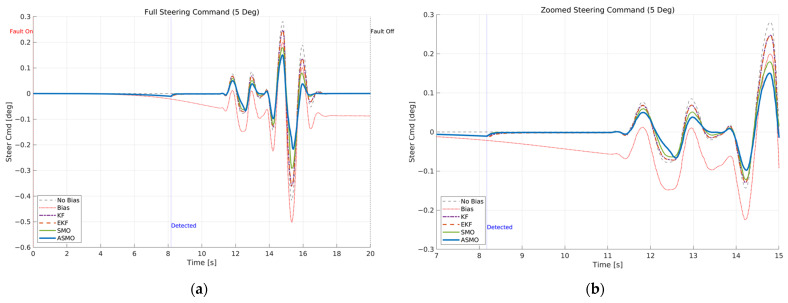
Controller steering command responses under Scenario 3: (**a**) Overall steering command during the double lane change maneuver; (**b**) Enlarged view of the steering command behavior during the drift-active interval.

**Figure 27:**
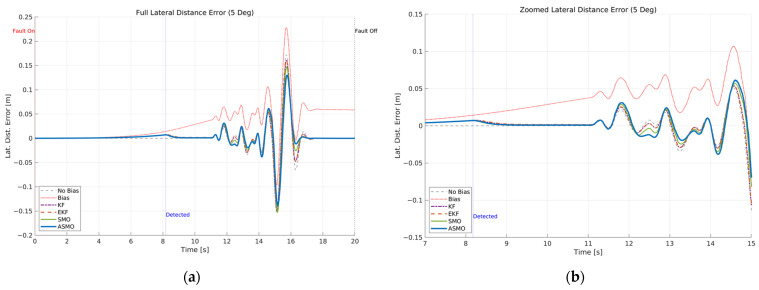
Lateral distance error under Scenario 3: (**a**) Overall lateral error during the double lane change maneuver; (**b**) Enlarged view highlighting accumulated tracking deviation caused by the drift fault.

**Figure 28:**
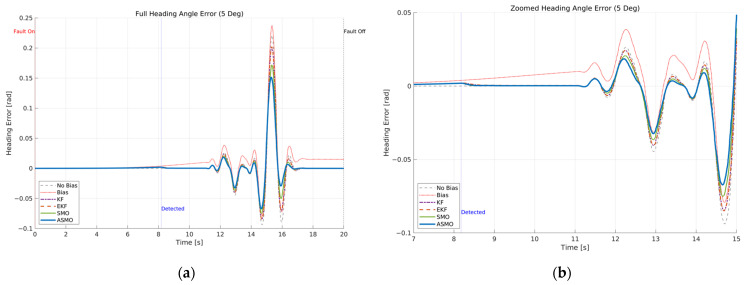
Heading angle error under Scenario 3: (**a**) Overall heading error response; (**b**) Enlarged transient response during the drift-active interval.

Figure 25, Figure 26, Figure 27 and Figure 28 present a comprehensive evaluation of the control behavior and tracking performance under the drift-type steering actuator bias in Scenario 3 at 100 km/h. Figure 25 illustrates the actual steering input applied to the vehicle. In the uncompensated case, the gradually increasing bias introduces a progressive offset in the effective steering angle. As the drift evolves over time, the steering response deviates increasingly from the nominal profile, particularly during the high-curvature phase of the DLC maneuver. The KF- and EKF-based approaches partially reduce this deviation; however, residual offset remains due to limited adaptability to slowly varying fault dynamics. The SMO-based method further improves compensation but still exhibits slight discrepancies during peak steering transitions. In contrast, the proposed ASMO-based control maintains a steering input closely aligned with the nominal condition throughout the maneuver, effectively suppressing the accumulated drift effect.

Figure 26 shows the corresponding controller steering command. In the presence of drift, the uncompensated controller does not account for the time-varying bias, leading to progressive distortion of the control effort. The KF and EKF methods provide partial correction, while the SMO approach reduces the magnitude of deviation more effectively. The ASMO-based controller, however, adaptively adjusts the command signal to counteract the gradual bias increase, preventing excessive control effort and preserving smooth steering behavior.

The impact of this compensation on tracking performance is quantified in Figure 27 and Figure 28. Figure 27 presents the lateral distance error, and Figure 28 shows the heading angle error. In the uncompensated case, both errors exhibit progressive accumulation as the drift evolves, reflecting the cumulative effect of the time-varying actuator offset. Although KF, EKF, and SMO methods reduce the magnitude of these deviations, noticeable residual errors persist, especially during the lateral stages of the maneuver. The ASMO-based strategy significantly suppresses both lateral and heading error accumulation, maintaining responses closely aligned with the nominal case and ensuring stable convergence after peak curvature transitions.

Overall, the results demonstrate that the proposed adaptive observer effectively compensates for gradually evolving actuator faults. By dynamically adjusting its gains in response to the drift component, the ASMO preserves steering integrity, mitigates error accumulation, and maintains stable path tracking performance under slowly varying fault conditions.

**Figure 29:**
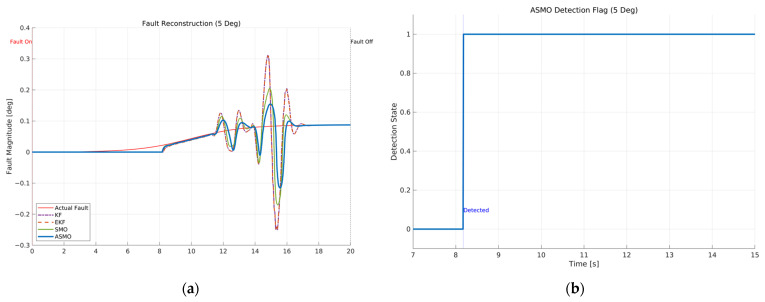
Fault reconstruction and detection performance under Scenario 3: (**a**) Comparison between actual drift fault magnitude and reconstructed estimates using KF, EKF, SMO, and ASMO observers; (**b**) ASMO-based fault detection flag indicating activation under gradually increasing bias.

Figure 29 presents the fault reconstruction and detection performance under the drift-type steering actuator bias in Scenario 3. As shown in Figure 29a, the actual fault magnitude increases gradually over time due to the drift component. The KF and EKF methods exhibit noticeable estimation mismatch and amplitude deviation during the dynamic phase of the maneuver, particularly when the steering dynamics become highly nonlinear. The SMO-based observer improves estimation accuracy; however, small discrepancies remain during peak steering transitions. In contrast, the proposed ASMO-based estimator closely follows the progressively increasing fault profile, maintaining bounded estimation error throughout the maneuver. This result demonstrates that the adaptive mechanism effectively tracks slowly varying actuator faults without excessive oscillation or divergence.

Figure 29b illustrates the corresponding detection flag generated by the ASMO-based logic. Despite the gradual nature of the drift faults, the detection signal transitions to the fault state once the accumulated deviation exceeds the threshold and remains stable thereafter. The absence of chattering indicates that the detection scheme successfully distinguishes between normal maneuver-induced dynamics and genuine actuator drift. This reliable detection enables timely activation of the FTC mechanism under slowly varying fault conditions.

**Figure 30:**
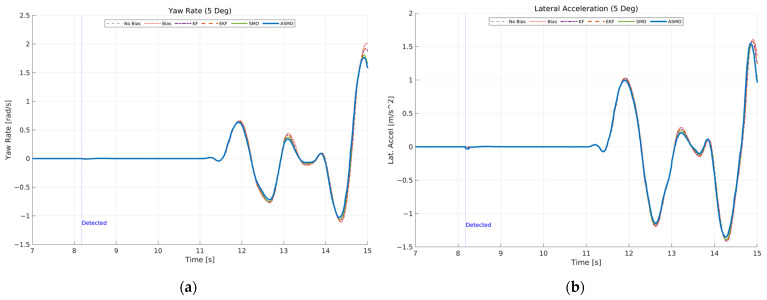
Transient vehicle dynamics response under Scenario 3: (**a**) Yaw rate response comparison; (**b**) Lateral acceleration response comparison.

Figure 30 presents the transient vehicle dynamic responses under the drift-type steering actuator bias in Scenario 3 at 100 km/h. The yaw rate and lateral acceleration responses are examined to evaluate the influence of gradually increasing actuator bias on vehicle stability. In the uncompensated case, the progressive drift in steering input alters the effective front wheel angle over time, leading to noticeable distortion in the yaw rate and lateral acceleration responses during the DLC maneuver. As the drift accumulates, peak amplitudes become increasingly asymmetric, and deviations from the nominal dynamic profile become more pronounced, particularly near the maximum curvature phase. The KF- and EKF-based approaches mitigate part of this dynamic distortion; however, residual discrepancies remain due to limited adaptability to a slowly varying fault component. The SMO-based method further improves damping characteristics, yet slight peak deviation persists during rapid steering transitions.

In contrast, the proposed ASMO-based control maintains yaw rate and lateral acceleration responses closely aligned with the nominal no-fault condition throughout the maneuver. Despite the gradual increase in actuator bias, the dynamic responses remain symmetric and well-damped, indicating that the adaptive observer effectively compensates for the time-varying drift. These results demonstrate that the proposed method preserves vehicle dynamic integrity while suppressing the accumulation effects associated with drift-type actuator faults.

**Figure 31:**
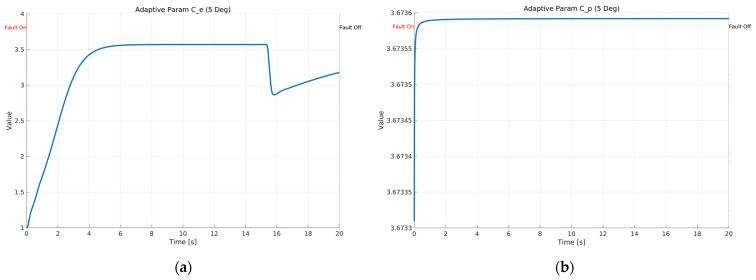
Time evolution of adaptive parameters under Scenario 3: (**a**) Adaptive parameter $C_{e}$ showing gradual gain adjustment in response to progressive bias drift; (**b**) Adaptive parameter $C_{\rho }$ exhibiting rapid convergence and bounded behavior throughout the maneuver.

Figure 31 illustrates the time evolution of the adaptive parameters $C_{e}$ and $C_{\rho }$ in the proposed ASMO under the drift-type steering actuator bias condition at 100 km/h. As shown in Figure 31a, the adaptive parameter $C_{e}$ increases progressively after fault injection, reflecting continuous gain adjustment in response observed in constant bias scenarios, the drift fault induces a smooth and sustained modification of the observer gain. During the high-curvature phase of the maneuver, a light re-adjustment is observed, indicating sensitivity of the adaptive law to changing vehicle dynamics. Importantly, the parameter remains bounded throughout the maneuver, confirming stable adaptation without divergence.

Figure 31b presents the evolution of $C_{\rho }$ which rapidly converges during the initial phase and then remains nearly constant despite the ongoing drift. The absence of oscillation or abrupt variation indicates that the adaptive mechanism selectively modifies gains according to their functional roles. While $C_{e}$ actively responds to the slow variation in the fault component, $C_{\rho }$ maintains robustness and stability of the observer structure.

Overall, the coordinated and bounded evolution of adaptive parameters demonstrates that the proposed ASMO can effectively track slowly varying actuator faults while preserving observer stability. This adaptive behavior directly contributes to the sustained tracking accuracy and dynamic stability observed in the trajectory and error responses under drift-type fault conditions.

**Figure 32:**
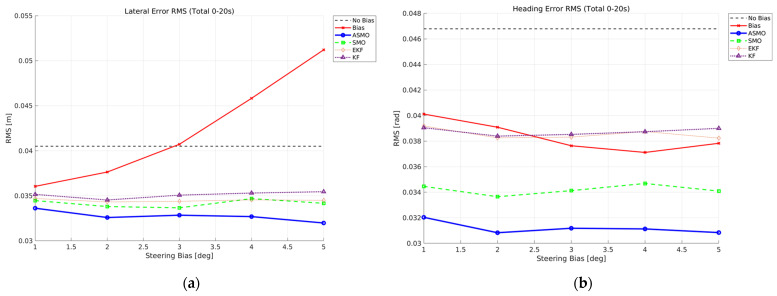
RMS tracking performance under Scenario 3: (**a**) Lateral distance RMS over the entire maneuver; (**b**) Heading angle RMS over the entire maneuver.

Figure 32 quantitatively evaluates the tracking robustness under drift-type steering actuator bias in Scenario 3. The RMS values of lateral distance and heading angle errors are computed over the entire maneuver for fault magnitudes ranging from 1 to 5 deg. In the uncompensated case, both lateral and heading RMS errors increase noticeably as the drift magnitude grows, reflecting the cumulative nature of the time-varying bias. Since the actuator offset gradually accumulates over time, larger drift amplitudes result in progressively greater deviation from the reference trajectory. The KF- and EKF-based approaches reduce the overall RMS levels compared to the uncompensated case; however, their performance shows limited improvement as the drift magnitude increases, indicating restricted adaptability to slowly varying fault components. The SMO-based method further lowers the RMS values, demonstrating improved disturbance rejection capability, yet residual error remains observable across all tested magnitudes.

In contrast, the proposed ASMO-based strategy consistently achieves the lowest RMS errors for both lateral and heading responses. Notably, the RMS variation with respect to increasing drift magnitude remains minimal compared to other methods, indicating strong robustness against accumulated actuator bias. These results confirm that the adaptive gain mechanism enables effective compensation of slowly varying faults while preserving overall tracking stability.

Table 4 summarizes the quantitative performance indices of fault detection and estimation under Scenario 3, where a drift-type steering actuator bias is introduced with magnitudes ranging from 1 to 5 deg. Compared with constant and intermittent fault cases, the detection delay under drift conditions is significantly longer. For a 1 deg drift fault, the detection delay is approximately 11.16 s, decreasing to approximately 8.18 s at 5 deg. This behavior is expected because the drift fault evolves gradually over time, requiring sufficient accumulation of deviation before exceeding the detection threshold. Nevertheless, the delay consistently decreases as the fault magnitude increases, indicating that larger drift amplitudes produce more distinguishable residual responses.

Importantly, the FAR and MDR remain zero across all tested magnitudes. This confirms that the proposed detection logic reliably discriminates between normal vehicle dynamics and slowly varying actuator faults without chattering or false triggering, even under gradual bias accumulation.

The improvement indices for lateral distance error and heading angle error further demonstrate the effectiveness of the FTC. As the drift magnitude increases, the improvement ratios grow steadily, reaching approximately 37.6% reduction in lateral error and approximately 18.4% reduction in heading error at 5 deg. These results indicate that the proposed adaptive framework becomes increasingly beneficial as the severity of the drift fault increases.

Overall, the results confirm that although drift-type faults require longer detection time due to their gradual evolution, the ASMO-based framework maintains reliable fault identification and provides substantial tracking performance recovery under slowly varying actuator bias conditions.

### 5.4. Additional Robustness Evaluation Under Parameter Variations

To evaluate the robustness of the proposed ASMO-based framework against modeling uncertainties, additional simulations were conducted under significant vehicle parameter perturbations. Specifically, the vehicle mass was reduced by 15% to represent payload variation, and the road friction coefficient was decreased from μ=1.0 to μ=0.6 to emulate low-friction conditions. These changes directly affect tire cornering stiffness and lateral force generation, thereby introducing substantial deviation from the nominal linearized vehicle model used for observer design.

**Figure 33:**
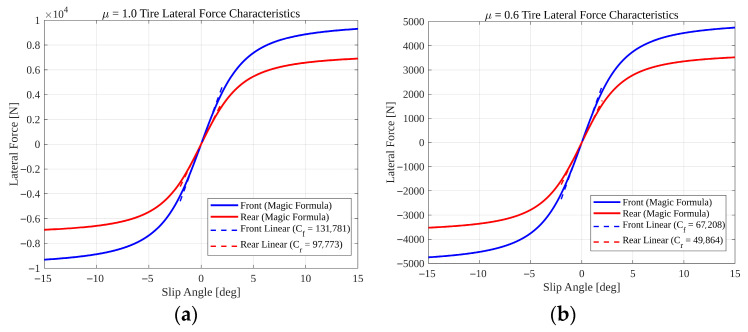
Tire lateral force characteristics under nominal and reduced friction conditions: (**a**) μ=1.0 (dry surface); (**b**) μ=0.6 (low-friction surface).

Figure 33 illustrates the tire lateral force characteristics under nominal (μ=1.0) and reduced-friction (μ=0.6) conditions. The nonlinear tire forces computed using the Pacejka Magic Formula are compared with linear tire approximations based on constant cornering stiffness values. Under nominal friction conditions, the linear tire model provides a reasonable approximation within the small slip angle region; however, noticeable deviation occurs in the saturation region where nonlinear tire dynamics dominate. When the friction coefficient is reduced to μ=0.6, both the peak lateral force and the effective slope around the origin decrease significantly. This reduction represents a change in effective cornering stiffness, directly influencing vehicle lateral dynamics. Despite these variations in tire force characteristics, the proposed ASMO-based observer was designed using a linearized vehicle model with fixed nominal cornering stiffness parameters. The robustness results presented in Section 5.4 demonstrate that the adaptive gain mechanism compensates for these modeling discrepancies, maintaining stable fault estimation and tracking performance even under reduced friction conditions. These observations confirm that the proposed framework tolerates variations in effective tire-road interaction properties without requiring explicit online cornering stiffness estimation or nonlinear observer redesign.

Table 5 summarizes the fault detection robustness of the proposed ASMO under significant vehicle parameter perturbations. Compared with the nominal parameter condition, the detection delay exhibits only a minor variation when the vehicle parameters are perturbed. Specifically, the delay remains within a comparable range (9.40 ms under nominal parameters and 8.18 ms under variation), indicating that the detection mechanism is not overly sensitive to model uncertainties. Importantly, both the FAR and MDR remain zero under all tested conditions. This confirms that the detection logic reliably discriminates between genuine actuator faults and dynamic changes induced by parameter perturbations. The absence of chattering or false triggering demonstrates that the residual-based threshold design maintains robustness even when the underlying vehicle dynamics are altered.

Overall, these results indicate that the proposed ASMO-based detection framework preserves stable and consistent fault identification performance despite significant variations in vehicle parameters.

**Figure 34:**
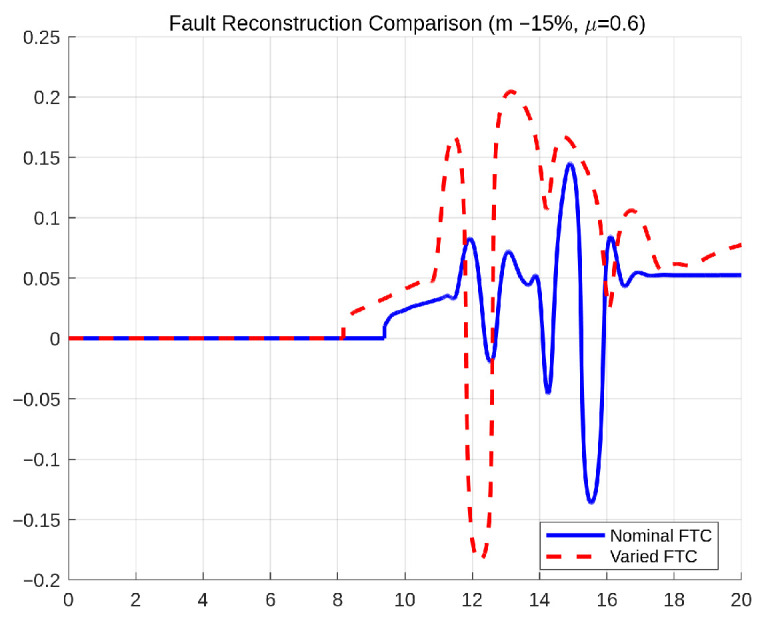
Fault Reconstruction comparison demonstrating stable estimation under significant vehicle parameter variations.

Figure 34 compares the fault reconstruction performance under nominal vehicle parameters and significant parameter perturbations (mass −15% and μ=0.6). Although slight amplitude differences are observed during highly dynamic phases of the maneuver, the estimated fault profile remains bounded and closely follows the actual drift behavior in both cases. The parameter variation introduces modeling discrepancies in both tire-road interaction characteristics and inertial vehicle dynamics. However, the adaptive gain mechanism of the ASMO compensates for these perturbations without causing divergence, excessive oscillation, or instability in the estimation process. The absence of significant estimation bias under perturbed conditions confirms that the observer does not rely heavily on precise parameter matching. These results further validate that the proposed framework maintains stable fault reconstruction performance even under combined inertial and friction uncertainties, supporting its applicability in realistic driving environments.

**Figure 35:**
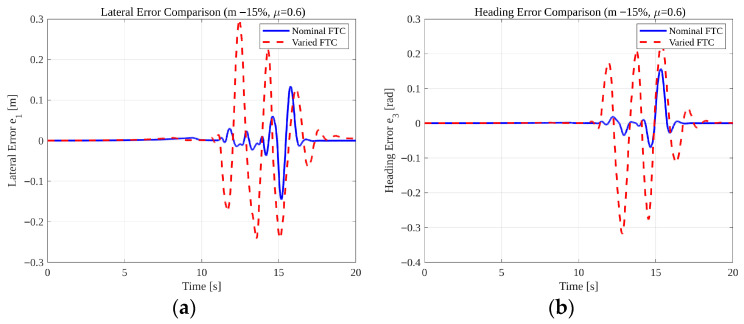
Tracking performance comparison under significant vehicle parameter variation: (**a**) Lateral distance error comparison; (**b**) Heading angle error comparison.

Figure 35 compares the tracking performance under nominal vehicle parameters and significant parameter variations (mass −15% and μ=0.6). As shown in both lateral and heading responses, parameter perturbations increase transient peak errors and introduce larger oscillatory behavior during the high-curvature phase of the maneuver. This degradation is expected because reduced friction lowers the effective cornering stiffness while mass reduction alters the vehicle’s inertial dynamics and natural frequency. The quantitative impact of these perturbations is summarized in Table 6. Under nominal conditions, the lateral and heading RMS errors remain at 0.0327 m and 0.031 rad, respectively. When both friction and mass are perturbed, the RMS values increase to 0.0916 m and 0.095 rad. Although this corresponds to noticeable percentage increases, the vehicle remains dynamically stable and the FTC mechanism continues to operate without divergence or loss of controllability. Peak lateral and heading errors also increase, yet remain bounded within physically reasonable limits for the given low-friction condition.

Importantly, this degradation reflects the inherent physical limitations imposed by reduced tire-road friction and altered inertial properties rather than instability of the observer or detection mechanism. As demonstrated in Table 5 and Figure 34, fault reconstruction and detection performance remain stable under the same perturbations. Therefore, while tracking accuracy decreases under severe parameter mismatch, the proposed ASMO-based framework preserves fault-tolerant functionality and maintains stable vehicle behavior without parameter retuning. These results confirm that the adaptive observer compensates effectively for actuator faults even when combined with substantial modeling uncertainties, supporting its applicability in realistic driving environments with varying payload and road conditions.

## 6. Conclusions and Future Work

This study presented an ASMO-based steering actuator fault detection and AFTC framework for high-speed autonomous vehicle path tracking. The proposed method was designed to address steering bias-type faults, including constant offset, intermittent activation, and gradual drift, which critically affect vehicle lateral stability at high speeds. A primary contribution of this work is the demonstration that adaptive gain tuning within a linearized vehicle modeling framework achieves accurate fault reconstruction without requiring online cornering stiffness estimation or nonlinear observer redesign. Although the ASMO is derived from a linear vehicle model, it effectively accommodates nonlinear tire behavior described by the Pacejka Magic Formula through bounded adaptive gain adjustment. A single set of observer and adaptive parameters was applied consistently across all fault scenarios, confirming inherent robustness without retuning.

Comprehensive validation using ISO 3888-1 [32] DLC maneuvers at 100 km/h demonstrated clear advantages over conventional observers. Compared with KF and EKF approaches, which exhibited limited adaptability to abrupt and slowly varying fault dynamics, the proposed ASMO achieved significantly improved fault reconstruction accuracy and faster convergence under highly transient conditions. Relative to the fixed-gain SMO, the ASMO reduced estimation chattering and improved transient responsiveness through adaptive gain modulation. Quantitatively, the proposed method achieved over 50% reduction in lateral RMS error and substantial heading error improvement under severe fault conditions, while maintaining zero false alarms and zero missed detections across constant, intermittent, and drift fault cases.

Robustness evaluation under vehicle parameter variations, including ±15% mass changes and reduced low-road friction conditions, confirmed stable detection delay, bounded estimation behavior, and limited degradation in tracking performance. These results indicate reliable operation under realistic modeling uncertainties and varying road conditions. By isolating the adaptive mechanism within the same observer family and comparing it against KF, EKF, and fixed-gain SMO under identical modeling assumptions, the study demonstrates that the observed improvements originate from adaptive gain regulation rather than structural model differences. Consequently, the proposed AFTC scheme restores vehicle trajectories to near-nominal performance without excessive control effort or increased computational complexity, making it suitable for real-time high-speed autonomous driving applications.

The present framework focuses on steering bias-type faults. Future work will extend the approach to composite actuator faults involving simultaneous bias and loss-of-effectiveness, as well as integrated sensor fault scenarios. In addition, experimental validation on a real autonomous vehicle platform will be pursued to further verify computational feasibility and robustness under real-world uncertainties.

## Figures and Tables

**Table 1:** Vehicle and dynamic model parameters for control and fault reconstruction.

Division	Symbol	Value	Unit
Mass	m	1800	[kg]
Yaw moment of inertia	$I_{z}$	2800	$[kg\cdot m^{2}$]
Distance from CG to front axle	$l_{f}$	1.15	[m]
Distance from CG to rear axle	$l_{r}$	1.55	[m]
Wheelbase	L	2.7	[m]
LQR Parameter	$q_{1}$	200.0	[-]
	$q_{3}$	350.0	[-]
	r	4000.0	[-]
Initial Adaptive Rho	$\rho_{init}$	2.0	[-]
Adaptive gain 1	$\gamma_{1}$	0.0043	[-]
Adaptive gain 2	$\gamma_{2}$	0.016	[-]
Error threshold	$\varepsilon_{fd}$	0.01	[-]
Detection threshold	$\delta_{th}$	0.015	[-]
Minimum injection bound	$\rho_{min}$	0.01	[-]

**Table 2:** Performance index comparison of fault detection and estimation for Scenario 1.

Degree	Delay_ms	FAR	MDR	$\mathrm{Improve}\;e_{y}$	$\mathrm{Improve}\;e_{\psi }$
1	606.3	0	0	11.263	26.523
2	156.1	0	0	15.812	26.948
3	106.36	0	0	25.652	28.04
4	86.179	0	0	33.326	29.944
5	76.562	0	0	41.162	32.809

**Table 3:** Performance index comparison of fault detection and estimation for Scenario 2.

Degree	Delay_ms	FAR	MDR	$\mathrm{Improve}\;e_{y}$	$\mathrm{Improve}\;e_{\psi }$
1	166.83	0	0	−6.941	7.545
2	96.266	0	0	8.98	4.772
3	66.239	0	0	27.694	8.8124
4	55.683	0	0	41.449	11.722
5	46.638	0	0	53.714	17.495

**Table 4:** Performance index comparison of fault detection and estimation for Scenario 3.

Degree	Delay_ms	FAR	MDR	$\mathrm{Improve}\;e_{y}$	$\mathrm{Improve}\;e_{\psi }$
1	11,616	0	0	6.734	20.144
2	10,686	0	0	13.422	21.125
3	9397	0	0	19.35	17.158
4	8677	0	0	28.671	16.13
5	8177.1	0	0	37.564	18.472

**Table 5:** Detection robustness of the proposed ASMO under significant vehicle parameter perturbations.

Condition	Delay_ms	FAR	MDR
Nominal	9.40	0	0
Variation	8.18	0	0

**Table 6:** Tracking performance degradation under low-friction and reduced-mass conditions.

Condition	LatRMS	LatRMS_Delta_pct	HeadRMS	HeadRMS_Delta_pct	LatPeak	HeadPeak
Nominal	0.032678	0	0.031	0	0.145	0.156
Variation	0.091649	180.461	0.095	204.67	0.294	0.318

## Data Availability

The data presented in this study are available on request from the corresponding author.

## Abbreviations

The following abbreviations are used in this manuscript:

FTC	Fault-tolerant control
AFTC	Active fault-tolerant control
SMO	Sliding mode observer
2-DOF	Two degrees of freedom
MPC	Model predictive control
ECU	Electronic control unit
LQR	Linear quadratic regulator
RLS	Recursive least squares
CG	Center of gravity
ARE	Algebraic Riccati equation
DLC	Double lane change
RMS	Root mean square
EKF	Extended Kalman filter
FAR	False alarm rate
MDR	Missed detection rate
